# Real-world landscape of drug-related ocular injuries: a retrospective pharmacovigilance study

**DOI:** 10.3389/fphar.2026.1731545

**Published:** 2026-01-21

**Authors:** Guangyao Li, Zhihui Song, Shuning Li, Jiawei Wang

**Affiliations:** 1 Department of Pharmacy, Beijing Tongren Hospital, Capital Medical University, Beijing, China; 2 Beijing Tongren Eye Center, Beijing Key Laboratory of Ophthalmology and Visual Science, Beijing Tongren Hospital, Capital Medical University, Beijing, China

**Keywords:** adverse drug events, FAERS database, ocular injury, pharmacovigilance, signal detection

## Abstract

**Objective:**

This study aimed to detect risk signals of drug-related ocular injuries, delineate their real-world epidemiological features, and provide evidence-based guidance for safe clinical use by utilizing the US Food and Drug Administration Adverse Event Reporting System (FAERS).

**Methods:**

Adverse event reports classified under “Eye disorders” (System Organ Class, SOC) were extracted from the FAERS database covering Q1 2004 to Q4 2024. Disproportionality analyses were employed to identify drug-ocular injury associations, and the time-to-onset (TTO) of adverse reactions was analyzed using Weibull distribution modeling.

**Results:**

A total of 1,242,518 reports from 832,314 patients were included, with females accounting for 60.56% and elderly patients (≥65 years) for 21.63%. Serious outcomes comprised 62.80% of the reports. In total, 2,696 primary suspect drugs were identified, of which 359 met the signal detection criteria. High-risk drug categories included sensory organ drugs (ATC: S class, ROR = 4.93) and antineoplastic/immunomodulating agents (ATC: L class, most reports but ROR = 0.84). The top three drugs by signal strength were brolucizumab (ROR = 132.15), macrogol 400 (ROR = 117.96), and cenegermin (ROR = 60.19). The most common ocular injury types were blurred vision (121,517 cases), visual impairment (113,320 cases), and cataract (51,826 cases). TTO analysis indicated that most drugs exhibited an “early failure type” (β < 1), such as dupilumab (β = 0.68); only two drugs exhibited a random failure type.

**Conclusion:**

The risk of drug-related ocular injuries is primarily associated with sensory organ drugs and biologics, with the greatest risk occurring during the early treatment phase. Clinical monitoring should prioritize female and elderly patients, especially regarding ocular symptoms at the onset of drug therapy, to strengthen pharmacovigilance and inform personalized medication.

## 1 Introduction

Adverse drug reactions (ADRs) represent a global public health concern threatening patient safety, contributing significantly to hospitalization and mortality rates (Alhawass et al., 2014; Giardina et al., 2018). Among ADRs, drug-related ocular disorders warrant particular attention due to their insidious symptoms and potential for permanent visual impairment (Slattery et al., 2014). The unique anatomical and physiological characteristics of ocular tissues, such as the high permeability of the blood-ocular barrier, render them susceptible to the effects of both systemic and topical medications (Slattery et al., 2014). Clinical manifestations include dry eye syndrome, glaucoma, and retinopathies. For instance, antiepileptic drugs can induce retinopathy and optic nerve damage, immune checkpoint inhibitors may trigger uveitis and keratopathy, and systemic medications (e.g., antidepressants) are also associated with an increased risk of dry eye syndrome (Han et al., 2017; Hamed, 2019; Liu et al., 2020).

In recent years, the US Food and Drug Administration Adverse Event Reporting System (FAERS) database has emerged as a crucial tool for investigating drug-related ocular injuries (Ali et al., 2025; Chen et al., 2025; Chen et al., 2024; Huang et al., 2025; Le et al., 2024; Li et al., 2025a; Li et al., 2025b; Li et al., 2025c; Wu et al., 2025a; Wu et al., 2024a). Studies utilizing FAERS have revealed that ocular adverse events (AEs) associated with certain drugs (e.g., cataracts, lens opacities with cariprazine) were not documented in their prescribing information (Zhu et al., 2024). Dupilumab may induce ocular degenerative changes via the IL-4/IL-13 pathway (Bridgewood et al., 2022), and dexamethasone implants have been linked to severe complications such as corneal decompensation (Zhao et al., 2025). FAERS, with its large-scale data, real-time updates, and standardized analytical methods, offers advantages over traditional clinical studies in detecting rare or delayed signals (Wong et al., 2015; Hu et al., 2024; Jiang et al., 2024). However, existing research primarily focuses on individual drugs, specific drug classes (e.g., systemic medications), or particular ocular AEs, lacking a systematic, panoramic investigation encompassing all drug categories. For instance, while recent studies have provided valuable insights into ocular adverse reactions associated with systemic drugs using the FAERS database (Wu et al., 2025b), a comprehensive real-world profile of drug-related ocular injury across all drug categories—particularly regarding local ophthalmic agents—remains elusive. This gap hinders a holistic understanding of the risk landscape and the development of stratified monitoring strategies.

The objective of our study is to identify and analyze reports of drug-related ocular injury adverse events within the FAERS database over the past 2 decades. We aim to delineate the clinical characteristics of drug-related ocular injury in the real world, identify high-risk drugs associated with ocular damage, define the types of ocular injuries and their temporal patterns, and ultimately enhance the safety of pharmacotherapy.

## 2 Methods

### 2.1 Data source

Data for this study were sourced from the FAERS raw database downloaded from the official US FDA website (https://fis.fda.gov/extensions/FPD-QDE-FAERS/FPD-QDE-FAERS.html; FAERS Quarterly Data Extract Files). Publicly available since Q1 2004 and updated quarterly, we downloaded ASCII data packages covering Q1 2004 to Q4 2024. Each quarterly file contained seven data types: DEMO (patient demographic and administrative information; one record per event report), REAC (adverse events coded using Medical Dictionary for Regulatory Activities [MedDRA] terms), DRUG (drug/biologic information for reported medications), OUTC (patient outcomes for the event), RPSR (report sources), THER (drug therapy start and end dates), and INDI (indications for use/diagnoses coded using MedDRA terms). The breadth of FAERS data provides a robust basis for drug safety signal detection (Li et al., 2024).

### 2.2 Data cleaning

Data cleaning strictly followed FDA guidance documents. The deduplication process involved selecting the PRIMARYID, CASEID, and FDA_DT fields from the DEMO table. Reports were sorted by CASEID, FDA_DT, and PRIMARYID. For reports sharing the same CASEID, the report with the most recent FDA_DT was retained. If CASEID and FDA_DT were identical, the report with the largest PRIMARYID was kept (Zhao and Tao, 2024; Yin et al., 2022). Starting from Q1 2019, each quarterly data package included a deletion report list; reports listed therein were excluded after deduplication. From the FAERS database, we obtained data from 22,375,298 patient reports. Applying FDA deduplication rules, 3,761,306 duplicate reports from the same patients were removed, resulting in 18,613,992 unique patients and 55,357,463 AE reports.

### 2.3 Data standardization

AE names in FAERS are described using Preferred Terms (PTs) from MedDRA. As MedDRA is updated biannually (March and September), potentially altering PTs and their corresponding System Organ Classes (SOCs), we standardized FAERS PTs using the latest MedDRA dictionary version (MedDRA v27.1) and mapped them to the current SOCs for subsequent analysis. After extracting drug names from the FAERS database, we systematically mapped and standardized drug trade names, aliases, and generic names by referencing the World Health Organization Drug Dictionary (September 2024 version), converting them to International Nonproprietary Names for subsequent analysis. The core purpose of this process was to consolidate reports corresponding to the same active ingredient, thereby ensuring the accuracy and consistency of drug identification. The Anatomical Therapeutic Chemical (ATC) classification codes for drugs were annotated according to the World Health Organization ATC index.

### 2.4 Identification of drug-related ocular injuries

In this study, the adverse drug events were derived from the MedDRA v27.1, (http://www.meddra.org/). MedDRA PTs serve as unique identifiers for specific medical concepts (e.g., symptoms, signs, disease diagnoses). The terminology is hierarchically organized into five levels, grouping concepts via High-Level Terms (HLTs), High-Level Group Terms (HLGTs), and PTs. Ultimately, HLGTs are classified into SOCs based on etiology, manifestation site, or purpose, with each PT assigned a primary SOC. Utilizing this framework, we focused our analysis on PTs classified under the primary SOC of 'Eye disorders' in MedDRA v27.1. All PTs primarily classified under this SOC were included in the analysis. The complete list of specific PTs used is provided in Supplementary Table S1. We extracted AE reports involving ocular injury where a drug was designated as the “Primary Suspect” (PS). These reports were characterized by sex, age, time to onset, reporter type, reporting country, and outcome.

### 2.5 Statistical analysis

To assess associations between drugs and ocular injury AEs, we employed four established signal detection algorithms (Table 1; Supplementary Table S2): Reporting Odds Ratio (ROR), Proportional Reporting Ratio (PRR), Bayesian Confidence Propagation Neural Network (BCPNN), and Multi-item Gamma Poisson Shrinker (MGPS). Signal identification criteria were: (1) ROR: ≥3 reports and lower limit of 95% confidence interval (CI) >1; (2) PRR: ≥3 reports and lower limit of 95% CI >1; (3) BCPNN: Information Component (IC) lower bound (IC025) >0; and (4) MGPS: Empirical Bayes Geometric Mean lower bound (EBGM05) >2 and number of reports (a) >0. Meeting the threshold in all four methods indicated a potential drug-event association (Chen et al., 2024; Wu et al., 2025a; Wu et al., 2024a). Each method has specific strengths: ROR corrects for under-reporting bias, while PRR enhances specificity by comparing the reporting rate for the drug-event pair against others. BCPNN, based on Bayesian principles, integrates data from diverse sources and supports cross-validation, enhancing signal robustness. MGPS excels at identifying rare signals and managing sparse data. By combining these methods, we maximized detection coverage and validated findings from multiple perspectives, strengthening the assessment of drug-ocular injury associations. SAS software (version 9.4) was used for database processing and statistical analysis. Statistical significance was set at p < 0.05.

**TABLE 1:** Four-fold table for disproportionality measurement in drug-related ocular injury signal detection.

Exposure group	Drug-related AEs	Non-drug-related AEs	Total
Drug	a	b	a + b
Non-drug	c	d	c + d
Total	a + c	b + d	N = a + b + c + d

a: number of reports containing both the suspect drug and the suspect adverse event.

b: number of reports containing the suspect adverse event with other medications (except the drug of interest).

c: number of reports containing the suspect drug with other adverse events (except the event of interest).

d: number of reports containing other drugs and other adverse events.

### 2.6 Time-to-onset analysis

Time-to-onset (TTO) was defined as the interval between the AE occurrence date (EVENT_DT in DEMO file) and the drug start date (START_DT in THER file). Reports with inaccurate date entries, missing specific dates, or errors (e.g., EVENT_DT before START_DT) were excluded. TTO was characterized using medians, interquartile ranges (IQR), and Weibull shape parameters (WSP) (Mazhar et al., 2021). The Weibull distribution shape is described by scale (α) and shape (β) parameters. AE hazard decreasing over time characterizes an early failure type (β < 1 and 95% CI < 1). A constant AE hazard over time characterizes a random failure type (β ≈ 1 and 95% CI includes 1). An increasing AE hazard over time characterizes a wear-out failure type (β > 1 and 95% CI > 1).

## 3 Results

### 3.1 Patient baseline characteristics and ocular injury adverse events

A total of 832,314 patients experiencing ocular injury AEs were included, involving 1,242,518 reports. Gender distribution showed a significantly higher proportion of females (60.56%, n = 504,021) compared to males (30.18%, n = 251,228), with sex unspecified in 9.26% (n = 77,065). The median age was 57.00 years (IQR 42.00–70.00). Patients aged ≥65 years accounted for 21.63% (n = 180,008), while age was unspecified in the largest proportion (38.65%, n = 321,662) (Table 2).

**TABLE 2:** Demographic and outcome characteristics of adverse event reports.

Indicator	Number (%)
Gender	
Female (%)	504,021 (60.56)
Male (%)	251,228 (30.18)
Not Specified (%)	77,065 (9.26)
Age	
<18 (%)	29,961 (3.60)
18–44 (%)	113,971 (13.69)
45–64 (%)	186,712 (22.43)
≥65 (%)	180,008 (21.63)
NotSpecified (%)	321,662 (38.65)
Age (Quantitative)	
N (Missing)	510,652 (321,662)
Mean (SD)	54.44 (20.21)
Median (Q1,Q3)	57.00 (42.00.70.00)
Min,Max	0.00,947.00
Report year	
2004	9605 (1.15)
2005	12,298 (1.48)
2006	13,522 (1.62)
2007	13,681 (1.64)
2008	15,162 (1.82)
2009	16,755 (2.01)
2010	25,465 (3.06)
2011	26,470 (3.18)
2012	29,281 (3.52)
2013	35,555 (4.27)
2014	35,595 (4.28)
2015	50,057 (6.01)
2016	48,343 (5.81)
2017	52,526 (6.31)
2018	59,067 (7.10)
2019	61,143 (7.35)
2020	61,923 (7.44)
2021	60,668 (7.29)
2022	62,655 (7.53)
2023	64,832 (7.79)
2024	77,711 (9.34)
Reporter	
Consumer (%)	416,491 (50.04)
Lawyer (%)	15,738 (1.89)
Not Specified (%)	38,857 (4.67)
Other health-professional (%)	80,203 (9.64)
Pharmacist (%)	96,151 (11.55)
Physician (%)	184,874 (22.21)
Outcome	
Life-threatening (%)	20,708 (2.49)
Hospitalization - initial or prolonged (%)	148,981 (17.90)
Disability (%)	42,422 (5.10)
Death (%)	17,839 (2.14)
Congenital Anomaly (%)	4449 (0.53)
Required intervention to prevent permanent Impairment/Damage (%)	5789 (0.70)
Other (%)	425,819 (51.16)
Time-to-Onset	
0-30d (%)	129,323 (15.54)
31-60d (%)	14,124 (1.70)
61-90d (%)	8543 (1.03)
91-120d (%)	6232 (0.75)
121-150d (%)	4563 (0.55)
151-180d (%)	3844 (0.46)
181-360d (%)	15,217 (1.83)
360d<(%)	37,462 (4.50)
Missing or anomalous values (less than 0) (%)	613,006 (73.65)
Time-to-onset (Quantitative)	
N (Missing)	219,308 (613,006)
Mean (SD)	272.63 (802.01)
Median (Q1,Q3)	11.00 (0.00,161.00)
Min,Max	0.00,73052.0

Serious outcomes are multi-select, so the sum of cases does not equal the total number of serious reports.

The majority of reports originated from the United States (62.91%, n = 523,595), followed by Canada (5.27%, n = 43,857) and the United Kingdom (4.37%, n = 36,371). Regarding AE outcomes, 62.80% were classified as serious, including hospitalization (17.90%, n = 148,981), disability (5.10%, n = 42,422), death (2.14%, n = 17,839), and life-threatening events (2.49%, n = 20,708), congenital anomaly (0.53%, n = 4449), required intervention to prevent permanent impairment/damage (0.70%, n = 5789), and others (51.16%, n = 425,819) (Table 2).

The TTO analysis revealed a median TTO of 11.00 days (IQR 0.00, 161.00) and a mean of 272.63 days (SD = 802.01). However, 73.65% of TTO data were excluded due to missing or anomalous values, leaving only 26.35% valid data. Among valid data, 15.54% of events occurred within 0–30 days post-drug initiation, while only 4.50% occurred after 360 days (Table 2).

### 3.2 Drug signal detection

The reported ocular injury AEs involved 2,696 unique “Primary Suspect” drugs, of which 359 met the signal detection criteria. The full list of these drug-induced ocular injury-related positive signal drugs and their corresponding ATC codes is provided in Supplementary Table S3. Analysis by ATC classification (Table 3) revealed that drugs for sensory organs (ATC: S class) exhibited the highest signal strength (ROR = 4.93, 95% CI 4.91–4.95; PRR = 4.60, 95% CI 4.59–4.62), indicating the strongest association with ocular injury. Dermatologicals (ATC: D class), alimentary tract and metabolism (ATC: A class), respiratory system (ATC: R class), and antiparasitic products, insecticides and repellents (ATC: P class) also showed strong associations with ocular injury AEs (ROR>1, PRR>1, and IC025 >0). Antineoplastic and immunomodulating agents (ATC: L class) had the highest report count (410,071 reports) but a low ROR value (0.84, 95% CI 0.84–0.85), below the overall signal threshold (Table 3).

**TABLE 3:** Signal strength of drug-related ocular injuries by anatomical therapeutic chemical (ATC) class (sorted by number of reports in descending order).

ATC classifications	ATC code	Number of reports	ROR (95% CI)	PRR (95% CI)	IC (IC025)	EBGM (EBGM05)
Ntineoplastic and immunomodulating agents	L	410,071	0.84 (0.84.0.85)	0.85 (0.84.0.85)	−0.16 (-0.16)	0.90 (0.89)
Sensory organs	S	313,487	4.93 (4.91.4.95)	4.60 (4.59.4.62)	1.89 (1.88)	3.69 (3.68)
Dermatologicals	D	232,719	1.92 (1.92.1.93)	1.89 (1.88.1.90)	0.78 (0.78)	1.72 (1.71)
Alimentary tract and metabolism	A	219,439	1.39 (1.38.1.39)	1.37 (1.37.1.38)	0.39 (0.38)	1.31 (1.30)
Nervous system	N	177,608	0.71 (0.71.0.72)	0.72 (0.71.0.72)	−0.40 (-0.41)	0.76 (0.75)
Respiratory system	R	154,599	1.78 (1.77.1.79)	1.75 (1.74.1.76)	0.73 (0.72)	1.66 (1.65)
Cardiovascular system	C	110,852	0.94 (0.93.0.94)	0.94 (0.93.0.94)	−0.09 (-0.10)	0.94 (0.94)
Genito urinary system and sex hormones	G	96,414	0.93 (0.93.0.94)	0.94 (0.93.0.94)	−0.09 (-0.10)	0.94 (0.93)
Musculo-skeletal system	M	64,534	0.95 (0.94.0.96)	0.95 (0.94.0.96)	−0.07 (-0.08)	0.95 (0.95)
Antiinfectives for systemic use	J	63,599	0.85 (0.84.0.86)	0.85 (0.85.0.86)	−0.22 (-0.23)	0.86 (0.85)
Blood and blood forming organs	B	47,330	0.63 (0.62.0.63)	0.63 (0.62.0.64)	−0.63 (-0.65)	0.64 (0.64)
Systemic hormonal preparations, excl. Sex hormones and insulins	H	43,274	0.91 (0.90.0.92)	0.91 (0.90.0.92)	−0.13 (-0.14)	0.92 (0.91)
Various^a^	V	31,951	0.91 (0.90.0.92)	0.91 (0.90.0.92)	−0.13 (-0.15)	0.91 (0.90)
Antiparasitic products, insecticides and repellents	P	7350	1.64 (1.60.1.68)	1.62 (1.58.1.66)	0.69 (0.66)	1.62 (1.58)

^a^ refers to no clear ATC, classification.

If the same drug belongs to different ATC, classifications, the number of case reports will be repeatedly counted in each corresponding classification.

**FIGURE 1:**
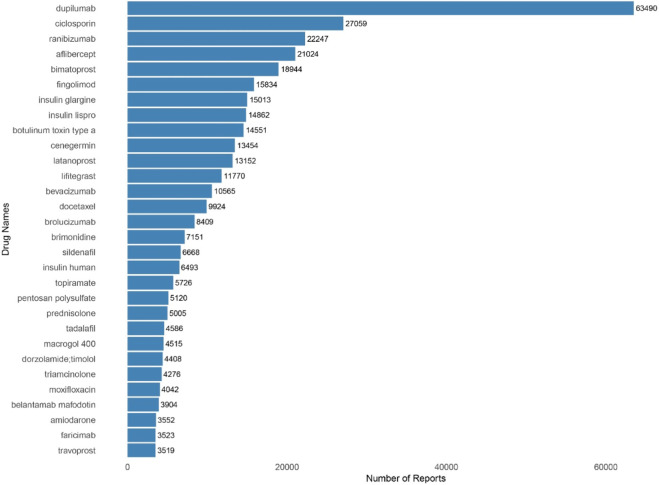
Top 30 primary suspect drugs for eye disorders (sorted by number of reports in Descending order).

Figure 1 and Table 4 displays the top 30 drugs with the most eye disorder reports. The top 30 drugs cover 14 ATC classes, including sensory organs (14 drugs), alimentary tract and metabolism (8 drugs), dermatologicals (7 drugs), and antineoplastic and immunomodulating agents (6 drugs). The top five drugs by report count are dupilumab (63,490 reports), ciclosporin (27,059 reports), ranibizumab (22,247 reports), aflibercept (21,024 reports), and bimatoprost (18,944 reports).Among specific drugs, brolucizumab (ROR = 132.15, 95% CI 126.61–137.94), macrogol 400 (ROR = 117.96, 95% CI 111.52–124.77), and cenegermin (ROR = 60.19, 95% CI 58.64–61.78) ranked highest in signal strength.

**TABLE 4:** Top 30 drugs with the most eye disorder reports (sorted by number of reports in descending order).

ATC code	Drug name	Number of reports	ROR (95% CI)	PRR (95% CI)	IC (IC025)	EBGM (EBGM05)
A16AX; D11AH; R01; R03DX	Dupilumab	63,490	4.23 (4.20.4.27)	3.96 (3.92.3.99)	1.93 (1.92)	3.80 (3.77)
L04AD; S01XA	Ciclosporin	27,059	7.71 (7.61.7.81)	6.72 (6.65.6.79)	2.72 (2.70)	6.59 (6.51)
S01LA	Ranibizumab	22,247	30.13 (29.62.30.65)	18.34 (18.15.18.53)	4.17 (4.15)	18.03 (17.72)
L01XX; S01LA	Aflibercept	21,024	30.22 (29.69.30.76)	18.37 (18.17.18.56)	4.18 (4.15)	18.07 (17.76)
D11AX; S01EE	Bimatoprost	18,944	41.29 (40.48.42.12)	21.83 (21.61.22.06)	4.43 (4.40)	21.52 (21.09)
L04AE	Fingolimod	15,834	2.59 (2.55.2.63)	2.50 (2.46.2.54)	1.31 (1.29)	2.48 (2.44)
A10AE	Insulin glargine	15,013	2.95 (2.90.2.99)	2.82 (2.78.2.87)	1.49 (1.46)	2.80 (2.76)
A10A; A10AB; A10AC; A10AD	Insulin lispro	14,862	3.44 (3.38.3.50)	3.26 (3.21.3.31)	1.69 (1.67)	3.23 (3.18)
A01AD; D11AA; D11AX; G04BD; M03AX; N02CX	Botulinum toxin type a	14,551	3.84 (3.77.3.90)	3.61 (3.55.3.66)	1.84 (1.81)	3.58 (3.52)
S01XA	Cenegermin	13,454	60.19 (58.64.61.78)	26.00 (25.72.26.29)	4.69 (4.65)	25.73 (25.07)
S01EE	Latanoprost	13,152	21.68 (21.23.22.14)	14.85 (14.65.15.06)	3.88 (3.85)	14.71 (14.40)
S01XA	Lifitegrast	11,770	28.22 (27.57.28.88)	17.58 (17.33.17.83)	4.12 (4.09)	17.42 (17.02)
L01FG; S01LA	Bevacizumab	10,565	2.30 (2.26.2.35)	2.24 (2.20.2.28)	1.15 (1.13)	2.23 (2.18)
L01CD	Docetaxel	9924	2.57 (2.52.2.62)	2.48 (2.43.2.53)	1.30 (1.27)	2.47 (2.42)
S01LA	Brolucizumab	8409	132.15 (126.61,137.94)	33.67 (33.31.34.04)	5.06 (5.02)	33.45 (32.05)
D11AX; S01EA; S01GA	Brimonidine	7151	16.48 (16.03.16.93)	12.25 (12.00.12.49)	3.61 (3.57)	12.18 (11.85)
C02KX; G04BE	Sildenafil	6668	3.05 (2.97.3.12)	2.91 (2.85.2.98)	1.54 (1.50)	2.90 (2.83)
A10A; A10AB; A10AC; A10AD; A10AE; A10AF	Insulin human	6493	3.25 (3.17.3.33)	3.09 (3.02.3.16)	1.62 (1.59)	3.08 (3.00)
N02CX; N03AX	Topiramate	5726	3.23 (3.14.3.32)	3.07 (3.00.3.15)	1.62 (1.58)	3.06 (2.98)
B01AB; C05BA; G04BX	Pentosan polysulfate	5120	35.97 (34.66.37.32)	20.19 (19.78.20.60)	4.33 (4.28)	20.11 (19.38)
A01AC; A07EA; C05AA; D07AA; H02AB; M02AX; R01AD; S01BA; S02BA; S03BA	Prednisolone	5005	2.12 (2.07.2.19)	2.07 (2.02.2.13)	1.05 (1.01)	2.07 (2.01)
C02KX; G04BE; G04CX	Tadalafil	4586	2.61 (2.54.2.69)	2.52 (2.45.2.59)	1.33 (1.29)	2.52 (2.44)
A06AD; S01XA	Macrogol 400	4515	117.96 (111.52,124.77)	32.62 (32.13.33.12)	5.02 (4.96)	32.51 (30.73)
S01ED	Dorzolamide; timolol	4408	34.24 (32.91.35.61)	19.64 (19.21.20.08)	4.29 (4.23)	19.57 (18.81)
A01AC; C05AA; D07AB; H02AB; R01AD; R03BA; S01BA	Triamcinolone	4276	4.77 (4.62.4.92)	4.40 (4.27.4.52)	2.13 (2.08)	4.38 (4.25)
D06AX; J01MA; S01AE; S03AA	Moxifloxacin	4042	2.88 (2.79.2.98)	2.77 (2.69.2.85)	1.47 (1.42)	2.76 (2.68)
L01FX	Belantamab mafodotin	3904	48.94 (46.75.51.23)	23.61 (23.11.24.13)	4.56 (4.49)	23.54 (22.49)
C01BD	Amiodarone	3552	2.08 (2.01.2.15)	2.03 (1.97.2.10)	1.02 (0.97)	2.03 (1.96)
S01LA	Faricimab	3523	26.73 (25.63.27.87)	16.96 (16.52.17.41)	4.08 (4.02)	16.91 (16.22)
S01EE	Travoprost	3519	25.19 (24.16.26.26)	16.34 (15.92.16.78)	4.03 (3.96)	16.30 (15.63)

Each drug may have different ATC, classifications, and all ATC, codes of the drug are listed in ATC, classifications.

### 3.3 Distribution of ocular injury types

Top 30 PTs for ocular injuries are shown in Table 5. The most common PTs for ocular injuries were vision blurred (121,517 reports), visual impairment (113,320 reports), cataract (51,826 reports), eye pain (46,937 reports), and eye irritation (45,436 reports). Ocular hyperaemia (39,886 reports), dry eye (39,479 reports) and blindness (35,863 reports) were also frequently reported.

**TABLE 5:** Top 30 MedDRA preferred terms (PTs) for ocular injuries (sorted by number of reports in descending order).

PT	Number of reports
Vision blurred	121,517
Visual impairment	113,320
Cataract	51,826
Eye pain	46,937
Eye irritation	45,436
Ocular hyperaemia	39,886
Dry eye	39,479
Blindness	35,863
Eye swelling	32,907
Visual acuity reduced	31,890
Eye disorder	29,085
Eye pruritus	27,097
Lacrimation increased	25,699
Diplopia	23,172
Glaucoma	17,281
Photophobia	16,290
Conjunctivitis	16,170
Blindness unilateral	12,616
Eye haemorrhage	12,204
Seasonal allergy	11,989
Uveitis	11,619
Mydriasis	11,475
Madarosis	11,201
Eyelid oedema	11,084
Macular degeneration	10,184
Eye infection	9857
Eyelid ptosis	8979
Ocular discomfort	8652
Eye discharge	8637
Vitreous floaters	8567

Table 6 lists the top 30 drug-PT pairs by signal strength (ROR value) from the drug-ocular injury signal detection. Pentosan polysulfate showed the strongest association with pigmentary maculopathy (ROR = 431,861, 95% CI 178,794–1,043,119), suggesting an extremely high risk. Moxifloxacin; triamcinolone was strongly associated with haemorrhagic occlusive retinal vasculitis (ROR = 58,840.7), and voretigene neparvovec with foveal degeneration (ROR = 58,148.2), indicating significant associations with specific retinal or corneal pathologies.

**TABLE 6:** Top 30 Drug-PT pairs with the highest ROR values for ocular injuries (sorted in descending order).

Drug name	Preferred term (PT) name	Number of reports	ROR (95% CI)	PRR (95% CI)	IC (IC025)	EBGM (EBGM05)
Pentosan polysulfate	Pigmentary maculopathy	426	431,861 (178794,1,043,119)	415,645 (172148,1,003,557)	12.24 (8.42)	4822.85 (1996.71)
Moxifloxacin; triamcinolone	Haemorrhagic occlusive retinal vasculitis	5	58,840.7 (22,613.3,153,106)	56,905.1 (22,473.9,144,087)	15.59 (1.30)	49,215.4 (18,914.1)
Voretigene neparvovec	Foveal degeneration	5	58,148.2 (20,828.9,162,333)	57,305.5 (20,753.8,158,233)	15.37 (1.23)	42,225.4 (15,125.3)
Besifloxacin	Diffuse lamellar keratitis	14	58,081.9 (26,315.2,128,196)	57,419.2 (26,118.9,126,229)	14.62 (2.97)	25,265.0 (11,446.8)
Cyclopentolate; phenylephrine; tropicamide	Punctate keratitis	7	42,432.9 (16,660.7,108,071)	26,800.1 (14,811.4,48,492.7)	14.70 (1.79)	26,555.8 (10,426.8)
Aprotinin; calcium chloride; factor i (fibrinogen); factor xiii (fibrin stabilising factor); thrombin	Conjunctival granuloma	3	35,637.7 (10,518.3,120,746)	35,184.7 (10,525.7,117,613)	14.90 (0.43)	30,595.5 (9030.12)
Pentosan polysulfate	Hereditary retinal dystrophy	7	34,170.3 (4203.63,277,762)	34,149.2 (4201.71,277,546)	12.06 (1.60)	4269.53 (525.24)
Mitomycin	Conjunctival filtering bleb leak	23	28,405.5 (14,124.0,57,127.6)	28,231.7 (14,058.2,56,694.6)	13.24 (3.82)	9680.09 (4813.22)
Ocriplasmin	Ciliary zonular dehiscence	5	24,407.1 (7060.92,84,366.3)	24,353.4 (7055.02,84,065.9)	13.57 (1.12)	12,177.2 (3522.84)
Lotilaner	Demodex blepharitis	4	24,080.8 (6461.17,89,748.8)	24,028.5 (6457.39,89,412.0)	13.70 (0.74)	13,349.6 (3581.87)
Calcium chloride; magnesium chloride; potassium; sodium acetate; sodium chloride; sodium citrate	Toxic anterior segment syndrome	411	22,935.6 (20,022.9,26,271.8)	18,666.5 (16,544.2,21,061.0)	13.39 (8.45)	10,700.2 (9341.39)
Carbachol	Iris haemorrhage	3	22,366.6 (6882.15,72,690.0)	21,967.2 (6894.03,69,996.4)	14.33 (0.48)	20,594.3 (6336.82)
Tetracaine	Aqueous fibrin	6	21,085.7 (8929.39,49,791.5)	20,738.2 (8892.47,48,363.6)	14.16 (1.63)	18,249.7 (7728.40)
Calcium chloride; magnesium chloride; potassium; sodium acetate; sodium chloride; sodium citrate	Aqueous fibrin	22	19,896.3 (11,365.2,34,831.2)	19,698.1 (11,287.3,34,376.4)	13.43 (3.79)	11,031.4 (6301.36)
Pentosan polysulfate	Retinal pigmentation	605	17,815.5 (15,041.0,21,101.9)	16,865.5 (14,268.4,19,935.3)	11.89 (8.87)	3784.71 (3195.29)
Carmellose; hypromellose	Conjunctival granuloma	3	14,934.4 (4425.18,50,401.7)	14,854.3 (4426.50,49,847.3)	13.66 (0.44)	12,916.9 (3827.37)
Belantamab mafodotin	Corneal epithelial microcysts	73	14,161.3 (9593.50,20,903.9)	14,021.4 (9511.50,20,669.6)	12.25 (5.76)	4883.10 (3308.03)
Calcium chloride	Dellen	4	13,989.4 (4505.99,43,431.9)	13,947.1 (4503.93,43,189.5)	13.35 (0.85)	10,460.6 (3369.36)
Ascorbic acid; betacarotene; tocopherol	Chemical burns of eye	5	13,585.2 (5424.59,34,022.6)	12,556.1 (5369.46,29,361.6)	13.59 (1.34)	12,370.9 (4939.72)
Mitomycin	Blebitis	18	13,320.4 (7040.65,25,201.3)	13,256.6 (7018.21,25,040.2)	12.77 (3.44)	6977.64 (3688.11)
Boric acid; pilocarpine; thiamine	Corneal opacity	4	13,136.3 (4317.72,39,965.9)	10,217.3 (4297.19,24,293.5)	13.31 (0.87)	10,183.5 (3347.18)
Ivermectin	Onchocerciasis	3	12,842.0 (1335.68,123,470)	12,839.0 (1335.54,123,425)	11.65 (0.06)	3210.49 (333.92)
Amantadine	Opsoclonus	7	12,731.1 (3291.60,49,240.3)	12,722.3 (3290.26,49,192.6)	11.90 (1.66)	3817.38 (986.98)
Trypan blue	Corneal oedema	4	12,048.2 (3707.30,39,154.9)	8341.36 (3686.45,18,874.1)	13.02 (0.82)	8325.06 (2561.67)
Brolucizumab	Retinal perivascular sheathing	66	11,718.2 (7528.59,18,239.4)	11,649.2 (7490.05,18,117.8)	11.76 (5.58)	3470.67 (2229.79)
Carbachol	Anterior chamber fibrin	4	11,639.4 (4245.94,31,907.3)	11,362.3 (4242.23,30,432.8)	13.42 (0.99)	10,983.6 (4006.71)
Netarsudil	Blepharitis allergic	9	11,566.9 (5291.32,25,285.5)	11,516.4 (5280.83,25,114.8)	12.98 (2.27)	8061.77 (3687.88)
Besifloxacin	Corneal flap complication	3	11,306.4 (3186.61,40,116.4)	11,278.8 (3186.70,39,919.3)	13.14 (0.38)	9023.22 (2543.11)
Netarsudil	Cornea verticillata	59	11,256.3 (8290.49,15,283.1)	10,933.9 (8103.57,14,752.9)	12.92 (5.47)	7771.96 (5724.20)
Belantamab mafodotin	Keratopathy	771	10,819.2 (9696.23,12,072.2)	9690.38 (8729.35,10,757.2)	12.04 (9.22)	4225.48 (3786.90)

### 3.4 Time-to-onset (TTO) analysis of adverse events

The TTO analysis based on the Weibull distribution is summarized in Table 7. The vast majority of drugs belonged to the Early Failure Type (β < 1 and 95% CI excludes 1), such as dupilumab (β = 0.68, 95% CI 0.66–0.69) and ciclosporin (β = 0.44, 95% CI 0.42–0.46), indicating the highest risk during the initial treatment period. Botulinum toxin type A had a median TTO of only 1 day, and ciclosporin had a median TTO of 0 days, suggesting some events occurred on the day of administration. The Random Failure Type (β ≈ 1 and 95% CI includes 1) included only belantamab mafodotin (β = 0.93, 95% CI 0.87–1.00) and pentosan polysulfate (β = 1.03, 95% CI 0.91–1.16). The former showed no significant time-dependence in risk, while the latter slightly exceeded one but the CI included 1, warranting cautious interpretation regarding long-term risk.

**TABLE 7:** Time-to-onset analysis using weibull distribution modeling for top 30 drugs (sorted by number of cases in descending order).

Durg name	Number of cases	TTO (days) Median (IQR)	Scale parameter α	95% CI	Shape parameter β	95% CI	Failure type
Dupilumab	4862	29.00 (0.00,150.00)	156.34	148.32–164.81	0.68	0.66–0.69	Early failure
Fingolimod	3910	37.50 (0.00,199.00)	201.44	188.65–215.10	0.59	0.57–0.60	Early failure
Aflibercept	3215	63.00 (1.00,435.00)	280.61	261.22–301.43	0.58	0.56–0.60	Early failure
Botulinum toxin type a	3154	1.00 (0.00.6.00)	14.17	12.81–15.67	0.49	0.47–0.50	Early failure
Ranibizumab	2892	67.00 (2.00,273.00)	200.1	187.00–214.11	0.63	0.61–0.65	Early failure
Docetaxel	2540	63.00 (18.50,265.00)	152.58	144.17–161.47	0.76	0.73–0.78	Early failure
Ciclosporin	1857	0.00 (0.00.16.00)	69.56	59.19–81.74	0.44	0.42–0.46	Early failure
Bevacizumab	1617	7.00 (0.00,107.00)	90.57	80.58–101.79	0.52	0.50–0.54	Early failure
Cenegermin	1513	4.00 (1.00.22.00)	28.42	25.17–32.10	0.51	0.49–0.53	Early failure
Bimatoprost	1408	1.00 (0.00.30.00)	38.84	33.30–45.29	0.45	0.43–0.47	Early failure
Brolucizumab	1302	35.00 (8.00.86.00)	78.77	73.17–84.80	0.85	0.81–0.89	Early failure
Latanoprost	1203	61.00 (0.00,731.00)	559.18	493.57–633.51	0.57	0.54–0.60	Early failure
Lifitegrast	873	0.00 (0.00.4.00)	29.05	23.30–36.22	0.52	0.49–0.57	Early failure
Naphazoline; pheniramine	824	0.00 (0.00.0.00)	38.4	23.23–63.48	0.35	0.31–0.39	Early failure
Topiramate	801	9.00 (3.00.30.00)	53.35	44.71–63.66	0.46	0.44–0.48	Early failure
Tadalafil	772	7.00 (0.00,161.00)	135.16	111.33–164.09	0.46	0.43–0.49	Early failure
Sildenafil	764	30.00 (0.00,609.50)	452.6	383.74–533.82	0.55	0.51–0.59	Early failure
Insulin glargine	757	181.00 (2.00,762.00)	560.8	490.52–641.16	0.63	0.59–0.67	Early failure
Moxifloxacin	752	0.00 (0.00.4.00)	16.34	13.02–20.50	0.48	0.45–0.52	Early failure
Insulin lispro	626	366.00 (3.00,1795.00)	1215.49	1054.52–1401.03	0.65	0.60–0.70	Early failure
Brimonidine	597	0.00 (0.00.5.00)	76.74	55.70–105.71	0.41	0.37–0.45	Early failure
Faricimab	560	50.00 (1.00,142.50)	118.86	105.73–133.63	0.84	0.78–0.91	Early failure
Insulin human	532	1445.50 (17.00,4017.00)	2763.25	2439.08–3130.51	0.79	0.73–0.86	Early failure
Belantamab mafodotin	414	35.00 (19.00.59.00)	62.78	56.01–70.38	0.93	0.87–1.00	Random failure
Triamcinolone	372	1.00 (0.00.6.00)	30.72	21.42–44.06	0.38	0.35–0.42	Early failure
Prednisolone	367	2.00 (0.00.14.00)	47.75	33.85–67.36	0.41	0.37–0.45	Early failure
Pentosan polysulfate	359	269.00 (0.00,3344.00)	3313.91	2883.14–3809.03	1.03	0.91–1.16	Random failure
Macrogol 400	265	0.00 (0.00.1.00)	6.72	4.36–10.37	0.56	0.48–0.64	Early failure
Travoprost	240	1.00 (0.00.54.00)	71.06	48.80–103.48	0.45	0.40–0.50	Early failure
Dorzolamide; timolol	238	1.00 (0.00.70.00)	169.97	111.47–259.18	0.43	0.38–0.49	Early failure

## 4 Discussion

By analyzing 1,242,518 reports of ocular injury AEs in the FAERS database from 2004 to 2024, this study delineates the epidemiological characteristics, high-risk drugs, and temporal risk patterns of drug-related ocular injury, providing crucial evidence-based insights for clinical medication safety. Compared to the recent study by Wu et al., which focused solely on ocular adverse reactions caused by systemic medications (Wu et al., 2025b), this study is the first to conduct a systematic panoramic analysis of all drug categories (including both topical and systemic agents) in FAERS. We not only confirmed the risks associated with some systemic drugs but also discovered that local ophthalmic agents (ATC: S class) exhibit the highest signal strength, thereby expanding our understanding of the risk spectrum for drug-induced ocular injury. Furthermore, through TTO analysis, this study quantified evidence of how risk changes over time, providing more refined guidance for clinical monitoring timing.

### 4.1 Patient demographics and risk factors

The study included a total of 832,314 patients, with a significantly higher proportion of females (60.56%) compared to males (30.18%). This finding is consistent with the results of multiple drug adverse reaction monitoring studies (Mao et al., 2024; Wu et al., 2024b). This phenomenon may be influenced by multiple factors. At the physiological level, sex differences can affect drug-metabolizing enzyme activity; females often exhibit higher plasma drug concentrations and prolonged elimination times, making them more susceptible to reaching toxicity thresholds (Zucker and Prendergast, 2020). Furthermore, the regulatory role of estrogen on the ocular surface may render female ocular tissues more sensitive to certain drugs, e.g., antihistamines, beta-blockers (Grasso et al., 2021). At the behavioral level, females generally demonstrate greater willingness and engagement in seeking healthcare, which may increase the likelihood of detecting and reporting adverse events (Watson et al., 2019).

In addition, the median age was 57.00 years (IQR 42.00–70.00), with elderly patients (≥65 years) comprising 21.63% of the cohort. Consistent with this, Wu et al. reported a mean age of 56.2 years (median: 60 years, IQR: 44–72) in drug-related keratitis patients, highlighting elevated risks in middle-aged and elderly populations (Wu et al., 2024b; Kozaru et al., 2023). These findings underscore age as a critical risk factor, likely driven by polypharmacy and age-related declines in hepatic/renal function, which alter drug metabolism in this group.

### 4.2 Sensory organ drugs: direct toxicity and mechanisms

Drugs for sensory organs (S class) exhibited the highest signal strength (ROR = 4.93), primarily due to their localized administration and pharmacological targets. As topical or intravitreal agents, they achieve high ocular tissue concentrations, bypassing systemic metabolism and directly exposing delicate ocular structures to drug effects (Koutsoviti et al., 2021). For example, the intravitreal injection drug brolucizumab (ROR = 132.15), used for wet age-related macular degeneration, showed high signal strength, necessitating vigilance for severe complications like retinal vasculitis and vitreous hemorrhage (Xiong et al., 2024). Similarly, topically applied prostaglandin analogs such as bimatoprost (ROR = 41.29) may induce conjunctival hyperemia, blepharitis, and eyelid lesions due to their long-term action on ocular surface tissues and impact on meibomian gland function (Manabe et al., 2020). The study by Williams et al. indicated that among patients using bimatoprost eye drops for long-term (4 years) treatment of ocular conditions, the incidence of blepharitis ranged from 2.6% to 5.1% (Williams et al., 2008). Furthermore, Smith et al. observed in a clinical study on eyelash growth that after daily application of 0.03% bimatoprost solution to the eyelid margin for 4 months, the incidence of adverse events such as eyelid lesions, conjunctival hyperemia, and dry eye all exceeded 2% (Smith et al., 2012). This heightened risk profile underscores that ocular ADRs predominantly stem from direct tissue exposure, the eye’s limited capacity for drug clearance, and immune privilege complexities. Vigilance remains critical, particularly for off-label uses altering drug-tissue contact.

### 4.3 Heterogeneity of antineoplastic and immunomodulating agents

Antineoplastic and immunomodulating agents (ATC: L class) had the highest number of reports (410,071 cases), yet the overall signal strength was low (ROR = 0.84). The high report count coupled with a low signal strength in ATC class L is a classic example of the dilution effect. This phenomenon arises from the massive patient population and the overshadowing of ocular symptoms by life-threatening systemic toxicities (e.g., myelosuppression) (Tanaka et al., 2022). Furthermore, the pharmacological heterogeneity within this class—ranging from highly specific ADCs to less toxic targeted therapies—further dilutes the average signal strength (Cui et al., 2025).

Traditional chemotherapeutic agents such as docetaxel (9,924 reports) exert cytotoxic effects by inhibiting tubulin polymerization, which may indiscriminately damage rapidly dividing epithelial cells on the ocular surface, leading to dry eye or corneal epithelial defects (Souza Monteiro de Araujo et al., 2022). In contrast, biologics such as the anti-CD38 antibody-drug conjugate (ADC) belantamab mafodotin (3,904 reports, ROR = 48.94) have a more specific toxicity mechanism: the drug binds to the CD38 antigen on corneal epithelial cells, internalizes, and releases its cytotoxic payload, resulting in the formation of corneal microcysts. This represents the ocular toxicity subtype with the strongest signal within ATC class L. (Mao et al., 2024; Farooq et al., 2020; Lu et al., 2023) This antigen-mediated targeted toxicity differs from the non-selective cytotoxicity of small-molecule drugs. Fingolimod (15,834 reports), another type of biologic acting as a sphingosine receptor modulator, may interfere with lymphocyte migration across the blood-ocular barrier, thereby inducing uveitis (Posarelli et al., 2011). It is also important to note that cancer patients often receive multi-drug combination therapy. Reported ocular damage may not be attributable to a single agent but rather the result of cumulative toxicity or interactions from multiple drugs, or it may be masked by the underlying disease or other more severe systemic toxicities. The spontaneous reporting nature of FAERS makes it challenging to control for this confounding effect of concomitant medications, highlighting the need for high-quality prospective studies to further explore this issue (Mao et al., 2024).

### 4.4 Dermatological drugs: local and systemic mechanisms

Dermatological drugs (ATC: D class) ranked third in this study with 232,719 reports and moderate signal strength (ROR = 1.92). Their association with ocular injury mainly stems from the dual mechanisms of local exposure and systemic immunomodulation. Botulinum toxin type A (14,551 cases) is commonly used to treat blepharospasm, hemifacial spasm, and various neuromuscular disorders of the head and neck. However, this treatment itself may cause blepharoptosis (drooping of the upper eyelid) and reduced blinking, leading to corneal epithelial defects and corneal ulcers (Nestor et al., 2021). Biologics such as dupilumab (63,490 cases), when used to treat atopic diseases, can precisely inhibit IL-4/IL-13 to improve symptoms such as asthma, but may cause ocular surface diseases (such as palpebral conjunctivitis, dry eye, etc.) and cytokine imbalance leading to abnormal function of ocular surface goblet cells due to immune response deviation (Bridgewood et al., 2022; Reji et al., 2022).

### 4.5 Common ocular injuries: clinical features and bias

Blurred vision (121,517 cases), visual impairment (113,320 cases), and cataract (51,826 cases) were the most common types of ocular injuries. Blurred vision and visual impairment, as the most common complaints of ADRs, may be because patients are more likely to perceive and report subjective experiences such as “inability to see clearly” rather than organic lesions requiring professional diagnosis (Karrer et al., 2019; Wu et al., 2025c). In contrast, cataract, as a clear clinical diagnosis, its high report volume reflects the direct association between drug exposure and organic damage. Long-term use of drugs such as glucocorticoids (e.g., prednisolone) and miotics (e.g., pilocarpine) can cause posterior subcapsular opacities by interfering with lens metabolism or inducing oxidative stress (Blomquist, 2011; Syed et al., 2021). These lesions require diagnosis by ophthalmic slit-lamp examination, so they are more likely to be actively reported by healthcare personnel. It is worth noting that the drug-relatedness of cataracts has a dose-time dependence, and regular ophthalmic screening should be implemented for long-term medication users (Al-Namaeh, 2024). This distinction between subjective symptoms and objective diagnoses is further highlighted by the composition of our data sources. Furthermore, consumer reports accounted for 50.04% of cases in this study, which may introduce reporting bias. Consumers tend to report easily perceptible subjective symptoms, whereas healthcare professionals are more likely to report based on objective diagnostic findings. This difference in reporting sources, along with factors such as patient education level and healthcare-seeking frequency, collectively influences the reporting frequency and patterns of different Preferred Terms. Future studies employing stratified analysis by reporter type could better elucidate this bias.

### 4.6 Time-to-onset patterns and monitoring

The TTO analysis revealed that most drugs exhibited an “early failure type” pattern (β < 1), such as dupilumab (β = 0.68) and ranibizumab (β = 0.63), indicating the highest risk within the first month post-initiation. This may relate to acute inflammatory responses or immune activation triggered by the drugs (Reji et al., 2022). Real-world studies have shown that ocular adverse events (such as conjunctivitis) associated with dupilumab in the treatment of atopic dermatitis primarily occur during the early treatment phase, especially within the first 4 months (Touhouche et al., 2021). Retrospective analysis revealed that 85.4% of severe intraocular inflammation events occurred within 5 days after ranibizumab injection (Souied et al., 2016). Only belantamab mafodotin (β = 0.93) and pentosan polysulfate (β = 1.03) showed a random failure pattern, suggesting sustained monitoring is required as risk lacks significant time-dependence (Farooq et al., 2020; Crivaro et al., 2019).

### 4.7 Study strengths and limitations

This study possesses two significant strengths. Firstly, in terms of data scale and methodology, it included over 1.2 million reports and employed four signal detection algorithms (ROR, PRR, BCPNN, MGPS) for cross-validation, ensuring the robustness of signals. Combining Weibull distribution analysis provided quantitative evidence of time-risk associations for pharmacovigilance. Secondly, regarding clinical guidance value, identifying high-risk categories (such as Sensory Organ drugs and biologics) through ATC classification and specific drug signals indicates that baseline ocular assessments and intensive early monitoring should be implemented for patients using these drugs.

However, the study also has some limitations. First, there are issues of data bias and incompleteness. As a spontaneous reporting system, FAERS has inherent limitations, including incomplete reporting and missing demographic information. In this study, gender was unspecified in 9.26% of cases, and age information was missing in 38.65% of cases. This may compromise an accurate assessment of risk characteristics in specific populations. Furthermore, time-to-event data were severely incomplete (73.65% missing TTO data), which limits the precise depiction of temporal patterns in drug-related ocular injuries, particularly hindering effective evaluation of late-onset risks associated with long-term medication use, such as cumulative chronic toxicity. Therefore, the TTO analysis based on the Weibull model in this paper primarily reflects the characteristics of cases with clearly documented time records, and its results, especially those concerning long-term risks, should be interpreted with caution. Future studies should integrate more comprehensive data sources to validate the findings. Second, the depth of mechanistic analysis is insufficient. Although high-risk drugs and temporal patterns were identified, this study lacks exploration into molecular mechanisms, such as blood-ocular barrier permeability and genetic susceptibility, which require further validation through basic experiments. Third, this study did not analyze the effects of dosage and combination therapy. Dosage information in FAERS reports is often recorded irregularly with a high missing rate, and it is impossible to accurately obtain patients' complete medication histories. Consequently, it is difficult to systematically analyze the impact of drug dosage, treatment duration, or concomitant medications on ocular injury, which may lead to the omission of important risk factors. Fourth, there is a limitation concerning the geographical representativeness of the data. The majority (62.91%) of the FAERS data utilized in this study originated from the United States. Consequently, the results may be influenced by local medication practices, healthcare reporting systems, and population genetic backgrounds. Therefore, caution should be exercised when extrapolating the conclusions to other ethnicities or regions. Future pharmacovigilance studies should integrate data from other regulatory agencies, such as those in the European Union and Japan, to verify the consistency of signals across different populations. Finally, constrained by the overall research objectives and the existing analytical framework of this study, stratified analyses based on reporter type, reporting country, or gender were not performed. We plan to prioritize stratified analysis in future research focusing on specific categories of drug-related ocular diseases, such as drug-related optic neuropathy.

### 4.8 Clinical risk management strategies

In clinical practice, several key strategies are recommended to mitigate risks. First, high-risk populations-including females, elderly patients, and those with underlying ocular diseases (e.g., diabetic retinopathy)-should undergo baseline ocular assessments before initiating relevant medications. Second, for drugs with “early failure type” patterns (e.g., dupilumab, ciclosporin), intensive monitoring of visual acuity, intraocular pressure, and fundus examinations during the first month of treatment is essential, while annual fundus screening should be incorporated for patients on long-term therapies (e.g., pentosan polysulfate). Lastly, patient education on proper topical eye drop administration is critical to minimize corneal irritation. Before initiating antineoplastic therapy, ocular contraindications should be assessed, and co-administration with other potentially eye-toxic drugs should be avoided.

## 5 Conclusion

This large-scale pharmacovigilance study delineates the real-world landscape of drug-related ocular injury, confirming the early treatment phase as the highest risk period and highlighting the need for focused attention on female and elderly patients. The findings provide evidence-based support for updating drug labeling, informing regulatory decisions, and guiding clinical monitoring strategies, ultimately contributing to enhanced medication safety. Future research should integrate mechanistic studies and prospective cohort analyses to further elucidate causal relationships in drug-related ocular toxicity, enabling more precise personalized pharmacotherapy.

## Data Availability

The original contributions presented in the study are included in the article/Supplementary Material, further inquiries can be directed to the corresponding author.
