# Supplementary material for: Real-world landscape of drug-related ocular injuries: a retrospective pharmacovigilance study
Source: Front Pharmacol. 2026 Jan 21;17:1731545. doi: 10.3389/fphar.2026.1731545 (PMC12868273; doi:10.3389/fphar.2026.1731545)
Supplement: Supplementary file 1 [file Supplementaryfile1.docx]

**Supplementary Table 1**. All Preferred Terms associated with drug-related ocular injuries included in the analysis

| **Preferred Term Code** | **Preferred Term** |
| --- | --- |
| 10058117 | Ocular icterus |
| 10077073 | Romana's sign |
| 10053227 | AIDS retinopathy |
| 10021629 | Inclusion conjunctivitis |
| 10076959 | Rhinocerebral mucormycosis |
| 10054775 | Viral corneal ulcer |
| 10078694 | Viral keratouveitis |
| 10062029 | Keratitis viral |
| 10010755 | Conjunctivitis viral |
| 10071005 | Viral uveitis |
| 10038915 | Retinitis viral |
| 10047646 | Vitreous abscess |
| 10074253 | Herpes zoster necrotising retinopathy |
| 10074252 | Herpes simplex necrotising retinopathy |
| 10082583 | Vitritis infective |
| 10074697 | Infective episcleritis |
| 10077918 | Infective scleritis |
| 10056500 | Infectious iridocyclitis |
| 10074698 | Infective iritis |
| 10075400 | Infective corneal ulcer |
| 10076672 | Infective keratitis |
| 10060833 | Infectious crystalline keratopathy |
| 10074700 | Infective uveitis |
| 10044269 | Toxocariasis |
| 10065119 | Necrotising herpetic retinopathy |
| 10069408 | Acanthamoeba keratitis |
| 10067817 | Acute haemorrhagic conjunctivitis |
| 10061788 | Corneal infection |
| 10010985 | Corneal abscess |
| 10078693 | Keratouveitis |
| 10010754 | Conjunctivitis tuberculous |
| 10010741 | Conjunctivitis |
| 10048843 | Cytomegalovirus chorioretinitis |
| 10084138 | Giant fornix syndrome |
| 10057182 | Periorbital cellulitis |
| 10051472 | Periorbital infection |
| 10052458 | Periorbital abscess |
| 10090458 | Ocular manifestation of Lyme disease |
| 10023637 | Lacrimal sac cellulitis |
| 10011844 | Dacryocystitis |
| 10079168 | Lacrimal gland abscess |
| 10011843 | Dacryocanaliculitis |
| 10023370 | Keratosis gonococcal |
| 10069166 | Blebitis |
| 10077286 | Post measles blindness |
| 10023349 | Keratoconjunctivitis measles |
| 10008779 | Choroid tubercles |
| 10008769 | Chorioretinitis |
| 10063202 | Mycotic endophthalmitis |
| 10030943 | Optic neuritis meningococcal |
| 10068612 | Candida retinitis |
| 10059449 | Candida endophthalmitis |
| 10030314 | Onchocerciasis |
| 10077477 | Staphylococcal blepharitis |
| 10021086 | Hypopyon |
| 10087989 | Demodex blepharitis |
| 10044325 | Trachoma |
| 10038910 | Retinitis |
| 10077496 | Varicella keratitis |
| 10016674 | Filariasis |
| 10063619 | Bacterial iritis |
| 10062028 | Keratitis bacterial |
| 10061784 | Conjunctivitis bacterial |
| 10056501 | Bacterial dacryocystitis |
| 10084936 | Bacterial endophthalmitis |
| 10078453 | Bacterial blepharitis |
| 10001257 | Adenoviral conjunctivitis |
| 10053991 | Inclusion conjunctivitis neonatal |
| 10049458 | Herpes simplex virus conjunctivitis neonatal |
| 10030861 | Ophthalmia neonatorum |
| 10015940 | Eye infection viral |
| 10088113 | Ocular vaccinia virus infection |
| 10015929 | Eye infection |
| 10015939 | Eye infection toxoplasmal |
| 10015935 | Eye infection gonococcal |
| 10015938 | Eye infection syphilitic |
| 10015905 | Eye abscess |
| 10015937 | Eye infection staphylococcal |
| 10065180 | Eye infection helminthic |
| 10015930 | Eye infection bacterial |
| 10015932 | Eye infection chlamydial |
| 10015933 | Eye infection fungal |
| 10062004 | Herpes ophthalmic |
| 10030865 | Ophthalmic herpes zoster |
| 10073938 | Ophthalmic herpes simplex |
| 10044819 | Tuberculosis of eye |
| 10007918 | Cellulitis orbital |
| 10064915 | Orbital infection |
| 10086753 | Ophthalmic cysticercosis |
| 10054762 | Eye infection intraocular |
| 10014801 | Endophthalmitis |
| 10063664 | Presumed ocular histoplasmosis syndrome |
| 10075264 | Oculoglandular syndrome |
| 10015988 | Eyelid infection |
| 10057211 | Eyelid folliculitis |
| 10000297 | Abscess of eyelid |
| 10015980 | Eyelid boil |
| 10010745 | Conjunctivitis chlamydial |
| 10054777 | Protozoal corneal ulcer |
| 10028518 | Mycotic corneal ulcer |
| 10062353 | Keratitis fungal |
| 10080825 | Conjunctivitis fungal |
| 10068613 | Fungal retinitis |
| 10038912 | Retinitis histoplasma |
| 10020377 | Hordeolum |
| 10085451 | Benedikt's syndrome |
| 10076985 | Bickerstaff's encephalitis |
| 10088816 | CANOMAD syndrome |
| 10078216 | CANVAS syndrome |
| 10085447 | Claude's syndrome |
| 10082594 | Foville syndrome |
| 10074972 | Irlen syndrome |
| 10084303 | Jeavons syndrome |
| 10085448 | Weber's syndrome |
| 10057375 | Balint's syndrome |
| 10053644 | IIIrd nerve disorder |
| 10021283 | IIIrd nerve paralysis |
| 10054202 | IIIrd nerve paresis |
| 10023110 | IVth nerve paralysis |
| 10053646 | VIth nerve disorder |
| 10047641 | VIth nerve paralysis |
| 10071044 | VIth nerve paresis |
| 10065836 | IVth nerve disorder |
| 10045178 | Tunnel vision |
| 10079769 | Cavernous sinus syndrome |
| 10020392 | Horner's syndrome |
| 10086833 | Supraorbital neuralgia |
| 10068097 | Raymond-Cestan syndrome |
| 10069714 | Ross syndrome |
| 10049567 | Miller Fisher syndrome |
| 10067462 | Millard-Gubler syndrome |
| 10088362 | Immune-mediated optic neuritis |
| 10019452 | Hemianopia |
| 10068101 | Vestibular nystagmus |
| 10029289 | Neurologic neglect syndrome |
| 10067348 | Chiasma syndrome |
| 10075539 | Delayed visual maturation |
| 10073653 | Visual perseveration |
| 10061411 | Visual pathway disorder |
| 10070920 | Visual cortex atrophy |
| 10080353 | Neuromyelitis optica pseudo relapse |
| 10077875 | Neuromyelitis optica spectrum disorder |
| 10030942 | Optic neuritis |
| 10084264 | Optic perineuritis |
| 10052784 | Retinal migraine |
| 10090043 | Opsoclonus |
| 10085806 | Myelin oligodendrocyte glycoprotein antibody-associated disease |
| 10019456 | Hemianopia homonymous |
| 10079491 | Microvascular cranial nerve palsy |
| 10067485 | Uhthoff's phenomenon |
| 10083324 | Ciliary ganglionitis |
| 10077820 | Quadrantanopia |
| 10058009 | Subacute myelo-opticoneuropathy |
| 10050122 | Ophthalmoplegic migraine |
| 10053854 | Opsoclonus myoclonus |
| 10091076 | Saccadic eye movement disorder |
| 10030867 | Ophthalmic migraine |
| 10029864 | Nystagmus |
| 10019455 | Hemianopia heteronymous |
| 10065562 | Crocodile tears syndrome |
| 10066418 | Tenon's cyst |
| 10057419 | Cataract operation complication |
| 10076662 | Closed globe injury |
| 10091138 | Vitreous wick syndrome |
| 10057436 | Vitreous injury |
| 10075399 | Persistent corneal epithelial defect |
| 10073679 | Traumatic iritis |
| 10018330 | Glaucoma traumatic |
| 10021281 | IIIrd nerve injury |
| 10023108 | IVth nerve injury |
| 10047639 | VIth nerve injury |
| 10066366 | Toxic anterior segment syndrome |
| 10070497 | Aqueous humour leakage |
| 10037756 | Radiation cataract |
| 10064714 | Radiation retinopathy |
| 10086652 | Iris tear |
| 10066373 | Floppy iris syndrome |
| 10057413 | Iris injury |
| 10008422 | Chemical burns of eye |
| 10054881 | Acquired pigmented retinopathy |
| 10082384 | Dysphotopsia |
| 10078321 | Pseudophakic bullous keratopathy |
| 10078211 | Pseudophakic glaucoma |
| 10071690 | Corneal flap complication |
| 10010984 | Corneal abrasion |
| 10091276 | Interface fluid syndrome |
| 10064713 | Radiation corneal injury |
| 10085832 | Corneal laceration |
| 10059166 | Keratorhexis |
| 10086034 | Corneal epithelial downgrowth |
| 10022120 | Injury corneal |
| 10081851 | Corneal graft failure |
| 10075519 | Eyelash injury |
| 10010733 | Conjunctival scar |
| 10010695 | Conjunctival abrasion |
| 10074785 | Conjunctival laceration |
| 10069165 | Conjunctival filtering bleb leak |
| 10079872 | Injury of conjunctiva |
| 10079837 | Conjunctival retraction |
| 10063938 | Posterior capsule rupture |
| 10063937 | Capsular block syndrome |
| 10073331 | Lenticular injury |
| 10086040 | Lens feathering |
| 10001907 | Amblyopia alcohol |
| 10076717 | Open globe injury |
| 10071697 | Periorbital haemorrhage |
| 10073500 | Lacrimal structure injury |
| 10071683 | Diffuse lamellar keratitis |
| 10063605 | Uveal prolapse |
| 10075397 | Bowman's membrane injury |
| 10081725 | Anterior capsular rupture |
| 10090878 | Glaucoma drainage device exposure |
| 10068148 | Uveitis-glaucoma-hyphaema syndrome |
| 10030949 | Optic pathway injury |
| 10030938 | Optic nerve injury |
| 10057430 | Retinal injury |
| 10071321 | Commotio retinae |
| 10016590 | Fibrin deposition on lens postoperative |
| 10088327 | Pupillary capture |
| 10007766 | Cataract traumatic |
| 10005185 | Blindness traumatic |
| 10014856 | Enophthalmos traumatic |
| 10042530 | Superficial injury of eye |
| 10049267 | Thermal burns of eye |
| 10061128 | Eye injury |
| 10073354 | Eye contusion |
| 10086801 | Eye abrasion |
| 10080964 | Ocular procedural complication |
| 10067683 | Eye laser scar |
| 10034544 | Periorbital haematoma |
| 10081061 | Intra-ocular injection complication |
| 10020923 | Hyphaema |
| 10027440 | Metallosis of globe |
| 10015971 | Eyeball avulsion |
| 10015948 | Eye luxation |
| 10076710 | Slipped extraocular muscle |
| 10017012 | Foreign body in eye |
| 10081521 | Eyelid abrasion |
| 10075018 | Eyelid contusion |
| 10069200 | Eyelid injury |
| 10082987 | Eyelid scar |
| 10076671 | Fat adherence syndrome |
| 10003267 | Arthritis reactive |
| 10077271 | Immunoglobulin G4 related disease |
| 10040767 | Sjogren's syndrome |
| 10086726 | Ocular torticollis |
| 10044686 | Trisomy 13 |
| 10044689 | Trisomy 22 |
| 10054935 | Aicardi's syndrome |
| 10081258 | BPES syndrome |
| 10081443 | Bergmeister's papilla |
| 10071755 | Blau syndrome |
| 10077624 | Bloch-Sulzberger syndrome |
| 10083269 | Bosch-Boonstra-Schaaf optic atrophy syndrome |
| 10064063 | CHARGE syndrome |
| 10083947 | De Barsy syndrome |
| 10079203 | Emanuel syndrome |
| 10080219 | Fraser syndrome |
| 10083933 | GM2 gangliosidosis |
| 10081685 | Kniest dysplasia |
| 10079437 | MYH9-related disease |
| 10088468 | Mainzer-Saldino syndrome |
| 10064583 | Marcus Gunn syndrome |
| 10079435 | Opitz-G/BBB syndrome |
| 10068032 | PHACES syndrome |
| 10082888 | RPE65 gene mutation |
| 10090250 | SHORT syndrome |
| 10083957 | Schmid Fraccaro syndrome |
| 10084074 | Senior-Loken syndrome |
| 10078901 | Winchester syndrome |
| 10081208 | Zhu-Tokita-Takenouchi-Kim syndrome |
| 10068783 | Alstroem syndrome |
| 10048734 | Phakomatosis |
| 10083306 | Galactosialidosis |
| 10059253 | Macrocornea |
| 10081275 | Monolid eyes |
| 10037150 | Pseudoxanthoma elasticum |
| 10057648 | Cyclopia |
| 10013799 | Duane's syndrome |
| 10056715 | Laurence-Moon-Bardet-Biedl syndrome |
| 10012565 | Developmental glaucoma |
| 10019819 | Hepato-lenticular degeneration |
| 10089596 | Sclerocornea |
| 10052452 | Osteoporosis-pseudoglioma syndrome |
| 10071775 | Hermansky-Pudlak syndrome |
| 10081541 | Iris hamartoma |
| 10052642 | Iris coloboma |
| 10066799 | Heterochromia iridis |
| 10063383 | Wagner's disease |
| 10088203 | Macular hypoplasia |
| 10062804 | Basal cell naevus syndrome |
| 10089751 | Muscle-eye-brain disease |
| 10048786 | Keratitis-ichthyosis-deafness syndrome |
| 10011005 | Corneal dystrophy |
| 10073484 | Lipodermoid tumour |
| 10086607 | Progressive encephalopathy, hypsarrhythmia and optic atrophy syndrome |
| 10036802 | Progressive external ophthalmoplegia |
| 10069402 | Wildervanck syndrome |
| 10087144 | Macrophthalmos |
| 10048804 | Kearns-Sayre syndrome |
| 10049066 | Cohen syndrome |
| 10009835 | Cockayne's syndrome |
| 10057855 | Hypotelorism of orbit |
| 10090531 | Cerulean cataract |
| 10070667 | Leber's congenital amaurosis |
| 10023636 | Lacrimal punctum agenesis |
| 10085252 | Lacrimo-auriculo-dento-digital syndrome |
| 10083192 | Dacryocystocoele |
| 10084376 | Split hand nystagmus syndrome |
| 10077917 | Lecithin-cholesterol acyltransferase deficiency |
| 10026829 | Marfan's syndrome |
| 10057402 | Choroidal coloboma |
| 10011385 | Cri du Chat syndrome |
| 10076031 | Mittendorf dot |
| 10067141 | Faciodigitogenital dysplasia |
| 10069760 | Norrie's disease |
| 10027974 | Morning glory syndrome |
| 10059199 | Anterior chamber cleavage syndrome |
| 10041513 | Spherophakia |
| 10064963 | Weill-Marchesani syndrome |
| 10009934 | Coloboma |
| 10073592 | Triple A syndrome |
| 10010050 | Colour blindness |
| 10082633 | Neurodegeneration with brain iron accumulation disorder |
| 10062940 | Neuropathy, ataxia, retinitis pigmentosa syndrome |
| 10090293 | Renal coloboma syndrome |
| 10062766 | Stargardt's disease |
| 10067159 | Septo-optic dysplasia |
| 10079368 | Tilted disc syndrome |
| 10062942 | Optic nerve hypoplasia |
| 10079967 | Optic disc pit |
| 10038824 | Retinal arteriovenous malformation |
| 10052643 | Retinal coloboma |
| 10038914 | Retinitis pigmentosa |
| 10018822 | Haemangioma of retina |
| 10085521 | Cone-rod dystrophy |
| 10083940 | Cone dystrophy |
| 10066128 | Distichiasis |
| 10063402 | Stickler's syndrome |
| 10042265 | Sturge-Weber syndrome |
| 10051713 | Ota's naevus |
| 10070872 | Persistent pupillary membrane |
| 10069203 | Waardenburg syndrome |
| 10048661 | Wyburn Mason's syndrome |
| 10078338 | Wolfram syndrome |
| 10083851 | Abetalipoproteinaemia |
| 10083858 | Aniridia-cerebellar ataxia-mental deficiency |
| 10002532 | Aniridia |
| 10002640 | Anophthalmos |
| 10005176 | Blindness congenital |
| 10007747 | Cataract congenital |
| 10061085 | Congenital vitreous anomaly |
| 10083496 | Congenital rubella syndrome |
| 10061530 | Congenital scleral disorder |
| 10057411 | Congenital iris anomaly |
| 10025412 | Macular dystrophy congenital |
| 10074554 | Congenital Horner's syndrome |
| 10011037 | Corneal opacity congenital |
| 10061059 | Congenital corneal anomaly |
| 10073654 | Congenital trichomegaly |
| 10069153 | Congenital myopia |
| 10024202 | Lens abnormality, congenital |
| 10062338 | Congenital lacrimal passage anomaly |
| 10011850 | Dacryostenosis congenital |
| 10010523 | Congenital lacrimal gland anomaly |
| 10089342 | Congenital cranial dysinnervation disorder |
| 10008763 | Chorioretinal degeneration congenital |
| 10057886 | Congenital choroidal anomaly |
| 10011761 | Cystic eyeball, congenital |
| 10001908 | Amblyopia congenital |
| 10074992 | Congenital astigmatism |
| 10059157 | Congenital visual acuity reduced |
| 10061528 | Congenital optic nerve anomaly |
| 10050706 | Retinopathy congenital |
| 10073470 | Congenital retinoblastoma |
| 10038938 | Retinoschisis congenital |
| 10038821 | Retinal anomaly congenital |
| 10019899 | Hereditary retinal dystrophy |
| 10086805 | Congenital anisocoria |
| 10050696 | Exophthalmos congenital |
| 10002947 | Aphakia congenital |
| 10042161 | Strabismus congenital |
| 10059159 | Congenital eye disorder |
| 10053202 | Posterior segment of eye anomaly congenital |
| 10002633 | Anomaly of orbit, congenital |
| 10051050 | Congenital eye naevus |
| 10061051 | Eye anterior chamber congenital anomaly |
| 10053877 | Congenital oculomotor apraxia |
| 10010562 | Congenital nystagmus |
| 10061146 | Congenital eyelid malformation |
| 10015996 | Eyelid ptosis congenital |
| 10056531 | Congenital epiblepharon |
| 10010559 | Congenital night blindness |
| 10064660 | Congenital lenticonus |
| 10049241 | Blepharophimosis congenital |
| 10014923 | Entropion congenital |
| 10057414 | Microcornea |
| 10027548 | Microphthalmos |
| 10010749 | Conjunctivitis gonococcal neonatal |
| 10065276 | Ocular albinism |
| 10063691 | Oculodentodigital dysplasia |
| 10057862 | Hypertelorism |
| 10088432 | Oculocerebrocutaneous syndrome |
| 10051707 | Oculocerebrorenal syndrome |
| 10052181 | Oculopharyngeal dystrophy |
| 10083860 | Ablepharon macrostomia syndrome |
| 10019864 | Hereditary choroidal dystrophy |
| 10019895 | Hereditary optic atrophy |
| 10011497 | Cryptophthalmos |
| 10088200 | Infantile neuroaxonal dystrophy |
| 10063396 | Usher's syndrome |
| 10081045 | Zika virus associated ocular birth defect |
| 10065273 | Prominent epicanthal folds |
| 10075540 | Silent sinus syndrome |
| 10085007 | Psychogenic blindness |
| 10076241 | Psychogenic visual disorder |
| 10059239 | Leukaemic retinopathy |
| 10052903 | Neoplasm of cornea unspecified malignancy |
| 10025871 | Malignant neoplasm of cornea |
| 10025861 | Malignant neoplasm of conjunctiva |
| 10026030 | Malignant neoplasm of lacrimal duct |
| 10026031 | Malignant neoplasm of lacrimal gland |
| 10025839 | Malignant neoplasm of choroid |
| 10026432 | Malignant neoplasm of retina |
| 10026183 | Malignant neoplasm of orbit |
| 10073086 | Iris melanoma |
| 10057412 | Iris neoplasm |
| 10066925 | Conjunctival primary acquired melanosis |
| 10081428 | Ciliary body melanoma |
| 10066384 | Conjunctival melanoma |
| 10067127 | Papilloma conjunctival |
| 10057406 | Conjunctival neoplasm |
| 10038956 | Retro-orbital neoplasm |
| 10057408 | Lacrimal duct neoplasm |
| 10087636 | Pleomorphic adenoma lacrimal gland |
| 10084088 | Lacrimal gland neoplasm |
| 10004316 | Benign neoplasm of cornea |
| 10004315 | Benign neoplasm of conjunctiva |
| 10004337 | Benign neoplasm of lacrimal duct |
| 10004338 | Benign neoplasm of lacrimal gland |
| 10004313 | Benign neoplasm of choroid |
| 10057424 | Benign neoplasm of optic nerve |
| 10004390 | Benign neoplasm of retina |
| 10004355 | Benign neoplasm of orbit |
| 10004325 | Benign neoplasm of eye |
| 10063707 | Benign neoplasm of eyelid |
| 10084635 | Choroidal osteoma |
| 10008773 | Choroid melanoma |
| 10070957 | Choroidal haemangioma |
| 10057405 | Choroid neoplasm |
| 10081431 | Uveal melanoma |
| 10073338 | Optic glioma |
| 10053645 | Optic nerve neoplasm |
| 10038878 | Retinal melanoma |
| 10057428 | Retinal melanocytoma |
| 10038916 | Retinoblastoma |
| 10057407 | Retinal neoplasm |
| 10090663 | Vasoproliferative retinal tumour |
| 10082449 | Ocular surface squamous neoplasia |
| 10086693 | Ocular melanoma |
| 10075324 | Ocular lymphoma |
| 10051045 | Eye naevus |
| 10071053 | Eye haemangioma |
| 10025910 | Malignant neoplasm of eye |
| 10061863 | Neoplasm of orbit |
| 10057416 | Ocular haemangiopericytoma |
| 10052448 | Ocular neoplasm |
| 10015831 | Extraocular retinoblastoma |
| 10007368 | Carcinoma in situ of eye |
| 10049724 | Metastases to eye |
| 10055099 | Ocular cancer metastatic |
| 10063706 | Malignant melanoma of eyelid |
| 10063693 | Malignant neoplasm of eyelid |
| 10057885 | Blepharal papilloma |
| 10071054 | Eyelid haemangioma |
| 10083592 | Eyelid seborrhoeic keratosis |
| 10050497 | Eyelid tumour |
| 10080691 | Eyelid naevus |
| 10068117 | Metastatic ocular melanoma |
| 10048908 | Seasonal allergy |
| 10011017 | Corneal graft rejection |
| 10077392 | Immune recovery uveitis |
| 10084816 | Ocular surface stem cell transplant rejection |
| 10087216 | Acute graft versus host disease in eye |
| 10083757 | Chronic graft versus host disease in eye |
| 10074563 | Graft versus host disease in eye |
| 10018706 | Graves' disease |
| 10043786 | Thyrotoxic crisis |
| 10051235 | Madarosis |
| 10059001 | Brow ptosis |
| 10030081 | Oculomucocutaneous syndrome |
| 10077355 | Pseudophakodonesis |
| 10084644 | Ocular implant exposure |
| 10072465 | Glassy eyes |
| 10088943 | Injection site foreign body sensation in eye |
| 10069837 | Eye complication associated with device |
| 10069034 | Tubulointerstitial nephritis and uveitis syndrome |
| 10051457 | Mikulicz's disease |
| 10052317 | Mikulicz's syndrome |
| 10078132 | MAGIC syndrome |
| 10004213 | Behcet's syndrome |
| 10018250 | Giant cell arteritis |
| 10071573 | Susac's syndrome |
| 10063354 | Charles Bonnet syndrome |
| 10073929 | IRVAN syndrome |
| 10023321 | Kayser-Fleischer ring |
| 10023482 | Koeppe nodules |
| 10087613 | Tenon's capsule thickening |
| 10003093 | Argyll-Robertson pupils |
| 10072729 | Delayed dark adaptation |
| 10007739 | Cataract |
| 10053150 | Leukocoria |
| 10005178 | Blindness day |
| 10074026 | Exfoliation glaucoma |
| 10074027 | Exfoliation syndrome |
| 10078951 | Exposure keratitis |
| 10083502 | Tessellated fundus |
| 10071641 | Bell's phenomenon |
| 10002500 | Angle closure glaucoma |
| 10043286 | Terrien's marginal degeneration |
| 10010744 | Conjunctivitis allergic |
| 10005149 | Blepharitis allergic |
| 10073286 | Pathologic myopia |
| 10047649 | Vitreous degeneration |
| 10047655 | Vitreous haemorrhage |
| 10047654 | Vitreous floaters |
| 10047650 | Vitreous detachment |
| 10071035 | Vitreomacular interface abnormal |
| 10047658 | Vitreous opacities |
| 10047651 | Vitreous disorder |
| 10072892 | Vitreous cyst |
| 10071181 | Vitreoretinal traction syndrome |
| 10063942 | Vitreous loss |
| 10047661 | Vitreous prolapse |
| 10077514 | Vitreous haze |
| 10066421 | Vitreal cells |
| 10052126 | Vitreous fibrin |
| 10071936 | Vitreous haematoma |
| 10047663 | Vitritis |
| 10057435 | Vitreous adhesions |
| 10038930 | Retinopathy hyperviscosity |
| 10051447 | Retinopathy haemorrhagic |
| 10085070 | Haemorrhagic occlusive retinal vasculitis |
| 10081000 | Vernal keratoconjunctivitis |
| 10005186 | Blindness unilateral |
| 10089147 | Monocular vision |
| 10044604 | Trichiasis |
| 10081899 | Hypotony maculopathy |
| 10074928 | Low luminance best-corrected visual acuity decreased |
| 10037508 | Punctate keratitis |
| 10068906 | Computer vision syndrome |
| 10000389 | Accommodation disorder |
| 10063452 | Arteriosclerotic retinopathy |
| 10030071 | Oculogyric crisis |
| 10017065 | Foster-Kennedy Syndrome |
| 10079469 | Malignant glaucoma |
| 10075386 | Malignant exophthalmos |
| 10089637 | Postictal blindness |
| 10074701 | Noninfective conjunctivitis |
| 10074696 | Noninfective chorioretinitis |
| 10074699 | Noninfective retinitis |
| 10069093 | Non-infectious endophthalmitis |
| 10081568 | Non-proliferative retinopathy |
| 10065311 | Paraneoplastic retinopathy |
| 10013036 | Diplopia |
| 10075567 | Dry age-related macular degeneration |
| 10013774 | Dry eye |
| 10048221 | Xerophthalmia |
| 10082802 | Disruption of the photoreceptor inner segment-outer segment |
| 10038926 | Retinopathy hypertensive |
| 10049935 | Lipaemia retinalis |
| 10030043 | Ocular hypertension |
| 10058646 | Arcus lipoides |
| 10054763 | Scleral thinning |
| 10061509 | Scleral degeneration |
| 10077825 | Scleral deposits |
| 10059096 | Scleral hyperaemia |
| 10050508 | Scleral haemorrhage |
| 10061510 | Scleral disorder |
| 10039694 | Scleral cyst |
| 10058954 | Scleromalacia |
| 10057432 | Scleral pigmentation |
| 10057431 | Scleral oedema |
| 10039696 | Scleral discolouration |
| 10052130 | Episcleral hyperaemia |
| 10015084 | Episcleritis |
| 10085163 | Scleral haematoma |
| 10039705 | Scleritis |
| 10037515 | Pupil fixed |
| 10063669 | Photoelectric conjunctivitis |
| 10034944 | Photokeratitis |
| 10079805 | Delayed light adaptation |
| 10051126 | Scleritis allergic |
| 10057380 | Allergic keratitis |
| 10007759 | Cataract nuclear |
| 10019370 | Heerfordt's syndrome |
| 10064729 | Dark circles under eyes |
| 10001902 | Amaurosis |
| 10053674 | Iris exfoliation |
| 10083516 | Iris discolouration |
| 10053677 | Iris transillumination defect |
| 10057418 | Iris haemorrhage |
| 10061254 | Iris disorder |
| 10053678 | Iridocorneal endothelial syndrome |
| 10022941 | Iridocyclitis |
| 10057410 | Hippus |
| 10067271 | Iridoplegia |
| 10022949 | Iris cyst |
| 10067684 | Iris bombe |
| 10022943 | Iridoschisis |
| 10053673 | Iris convex |
| 10053675 | Iris incarceration |
| 10049758 | Iris hyperpigmentation |
| 10063694 | Iris hypopigmentation |
| 10051450 | Iridocele |
| 10022942 | Iridodialysis |
| 10079167 | Ectropion uveae |
| 10022948 | Iris atrophy |
| 10065630 | Iris neovascularisation |
| 10061360 | Iris vascular disorder |
| 10022955 | Iritis |
| 10022945 | Iris adhesions |
| 10058357 | Iridodonesis |
| 10019099 | Halo vision |
| 10015290 | Erythropsia |
| 10036346 | Posterior capsule opacification |
| 10081651 | Posterior capsule neovascularisation |
| 10048851 | Necrotising scleritis |
| 10064997 | Necrotising retinitis |
| 10025409 | Macular degeneration |
| 10025425 | Maculopathy |
| 10085831 | Macular cherry-red spots |
| 10025416 | Macular opacity |
| 10060815 | Macular pseudohole |
| 10051058 | Macular hole |
| 10081199 | Macular telangiectasia |
| 10025407 | Macular cyst |
| 10065319 | Macular rupture |
| 10064697 | Epiretinal membrane |
| 10065534 | Macular ischaemia |
| 10091257 | Macular pigmentary changes |
| 10025415 | Macular oedema |
| 10075873 | Macular detachment |
| 10071392 | Macular fibrosis |
| 10025420 | Macular vasospasm |
| 10084571 | Macular thickening |
| 10063185 | Macular scar |
| 10048216 | Xanthopsia |
| 10065058 | Acquired corneal dystrophy |
| 10053990 | Dacryostenosis acquired |
| 10083194 | Dacryocystocoele acquired |
| 10011841 | Dacryoadenitis acquired |
| 10010051 | Colour blindness acquired |
| 10086462 | Acquired hypertrophy of the retinal pigment epithelium |
| 10056511 | Acquired epiblepharon |
| 10064659 | Acquired lenticonus |
| 10086444 | Acute macular neuroretinopathy |
| 10078300 | Acute myopia |
| 10048867 | Pseudophakia |
| 10075919 | Pseudomyopia |
| 10085407 | Pseudohypopyon |
| 10037112 | Pseudo-blepharoptosis |
| 10037141 | Pseudopapilloedema |
| 10072123 | Pseudostrabismus |
| 10069091 | Pseudoendophthalmitis |
| 10037147 | Pseudopterygium |
| 10023335 | Keratitis interstitial |
| 10083187 | Serous retinopathy |
| 10040114 | Serous retinal detachment |
| 10078509 | Heteronymous diplopia |
| 10042742 | Sympathetic ophthalmia |
| 10052122 | Dellen |
| 10011044 | Corneal scar |
| 10066968 | Corneal leukoma |
| 10084903 | Corneal leukoplakia |
| 10054760 | Corneal thinning |
| 10010996 | Corneal degeneration |
| 10023365 | Keratopathy |
| 10011026 | Corneal lesion |
| 10011000 | Corneal deposits |
| 10051558 | Corneal bleeding |
| 10011055 | Corneal touch |
| 10011039 | Corneal perforation |
| 10056476 | Corneal irritation |
| 10082599 | Corneal toxicity |
| 10053703 | Keratic precipitates |
| 10012532 | Detached Descemet's membrane |
| 10011035 | Corneal opacity |
| 10061453 | Corneal disorder |
| 10011022 | Corneal infiltrates |
| 10090966 | Corneal pseudoguttata |
| 10011013 | Corneal erosion |
| 10061787 | Corneal cyst |
| 10066970 | Corneal endothelial disorder |
| 10071681 | Corneal endothelial cell loss |
| 10062621 | Corneal endotheliitis |
| 10011048 | Corneal staphyloma |
| 10075404 | Corneal warpage |
| 10051559 | Corneal defect |
| 10023361 | Keratomalacia |
| 10011040 | Corneal pigmentation |
| 10080786 | Corneal epithelial wrinkling |
| 10011010 | Corneal epithelium defect |
| 10068144 | Corneal epithelial microcysts |
| 10087900 | Keratoneuralgia |
| 10052117 | Corneal decompensation |
| 10011033 | Corneal oedema |
| 10064489 | Corneal exfoliation |
| 10077604 | Cornea verticillata |
| 10055665 | Corneal neovascularisation |
| 10023332 | Keratitis |
| 10072138 | Limbal stem cell deficiency |
| 10070492 | Limbal swelling |
| 10071164 | Corneal thickening |
| 10055015 | Corneal hypertrophy |
| 10052118 | Corneal striae |
| 10044613 | Trichomegaly |
| 10048462 | Growth of eyelashes |
| 10058809 | Eyelash discolouration |
| 10058810 | Eyelash thickening |
| 10064121 | Eyelash hyperpigmentation |
| 10009185 | Ciliary muscle spasm |
| 10011719 | Cycloplegia |
| 10034051 | Pars plana cyst |
| 10009181 | Ciliary body degeneration |
| 10057415 | Ciliary body disorder |
| 10052129 | Ciliary hyperaemia |
| 10057417 | Ciliary body haemorrhage |
| 10090331 | Cyclodialysis cleft |
| 10011715 | Cyclitis |
| 10068351 | Ciliary zonular dehiscence |
| 10030863 | Ophthalmia nodosa |
| 10085433 | Conjunctival thinning |
| 10010712 | Conjunctival discolouration |
| 10010705 | Conjunctival degeneration |
| 10068819 | Conjunctival pallor |
| 10010708 | Conjunctival deposit |
| 10051625 | Conjunctival hyperaemia |
| 10010719 | Conjunctival haemorrhage |
| 10010725 | Conjunctival irritation |
| 10035060 | Pinguecula |
| 10062889 | Pingueculitis |
| 10053163 | Conjunctival opacity |
| 10061446 | Conjunctival disorder |
| 10010736 | Conjunctival ulcer |
| 10080291 | Conjunctival lymphangiectasia |
| 10010717 | Conjunctival follicles |
| 10072143 | Conjunctival telangiectasia |
| 10010716 | Conjunctival erosion |
| 10010703 | Conjunctival cyst |
| 10075398 | Conjunctivalisation |
| 10085660 | Conjunctival defect |
| 10010718 | Conjunctival granuloma |
| 10051631 | Conjunctival papillae |
| 10062276 | Conjunctival pigmentation |
| 10010726 | Conjunctival oedema |
| 10064132 | Conjunctivochalasis |
| 10085595 | Subconjunctival fibrosis |
| 10061783 | Conjunctival vascular disorder |
| 10010696 | Conjunctival adhesion |
| 10084034 | Conjunctival suffusion |
| 10052114 | Conjunctival bleb |
| 10028651 | Myopia |
| 10079959 | Myopic chorioretinal degeneration |
| 10080534 | Myopic traction maculopathy |
| 10069088 | Myopic disc |
| 10070549 | Lens discolouration |
| 10024214 | Lenticular opacities |
| 10061219 | Lens disorder |
| 10034798 | Phacolytic glaucoma |
| 10024216 | Lenticular pigmentation |
| 10024203 | Lens dislocation |
| 10087553 | Ciliary zonular weakness |
| 10065622 | Venous stasis retinopathy |
| 10018258 | Giant papillary conjunctivitis |
| 10030348 | Open angle glaucoma |
| 10056667 | Cogan's syndrome |
| 10065569 | Rhegmatogenous retinal detachment |
| 10066383 | Orbital apex syndrome |
| 10031045 | Orbital haemorrhage |
| 10082879 | Periorbital discomfort |
| 10082774 | Periorbital irritation |
| 10057425 | Periorbital disorder |
| 10087203 | Periorbital dermatitis |
| 10034546 | Periorbital pain |
| 10080722 | Periorbital inflammation |
| 10071682 | Periorbital fat atrophy |
| 10065121 | Periorbital fat herniation |
| 10056647 | Periorbital swelling |
| 10064996 | Ulcerative keratitis |
| 10051819 | Cyanopsia |
| 10091210 | Lupus retinopathy |
| 10036628 | Presbyopia |
| 10067427 | Rheumatoid scleritis |
| 10069930 | Lacrimal haemorrhage |
| 10023635 | Lacrimal passage granuloma |
| 10065118 | Inflammation of lacrimal passage |
| 10072718 | Lacrimal punctum enlarged |
| 10015552 | Eversion of lacrimal punctum |
| 10023627 | Lacrimal duct pigmentation |
| 10088552 | Tear film instability |
| 10051209 | Swollen tear duct |
| 10089330 | Lacrimal gland calcification |
| 10061525 | Lacrimal disorder |
| 10072746 | Lacrimal structural disorder |
| 10011848 | Dacryolith |
| 10061260 | Lacrimal cyst |
| 10023618 | Lacrimal atrophy |
| 10023632 | Lacrimal gland enlargement |
| 10060869 | Lacrimal mucocoele |
| 10091181 | Dysfunctional tear syndrome |
| 10023628 | Lacrimal fistula |
| 10038935 | Retinopathy sickle cell |
| 10006027 | Borderline glaucoma |
| 10023642 | Lacrimation decreased |
| 10023644 | Lacrimation increased |
| 10023643 | Lacrimation disorder |
| 10008585 | Chloropsia |
| 10033842 | Paralytic lagophthalmos |
| 10008786 | Choroidal haemorrhage |
| 10057403 | Choroidal infarction |
| 10008790 | Choroidal rupture |
| 10063970 | Choroidal effusion |
| 10008766 | Chorioretinal scar |
| 10063118 | Chorioretinopathy |
| 10061763 | Chorioretinal disorder |
| 10075124 | Chorioretinal folds |
| 10008783 | Choroidal detachment |
| 10060823 | Choroidal neovascularisation |
| 10068642 | Choroidal haematoma |
| 10008792 | Choroiditis |
| 10008785 | Choroidal dystrophy |
| 10052486 | Choroidal sclerosis |
| 10087281 | Immune-mediated scleritis |
| 10083069 | Immune-mediated uveitis |
| 10081123 | Autoimmune eye disorder |
| 10052119 | Cyclitic membrane |
| 10071570 | Ligneous conjunctivitis |
| 10052128 | Glare |
| 10007764 | Cataract subcapsular |
| 10058202 | Cystoid macular oedema |
| 10076660 | Cortical visual impairment |
| 10060742 | Endocrine ophthalmopathy |
| 10064930 | Age-related macular degeneration |
| 10072959 | Birdshot chorioretinopathy |
| 10056696 | Gaze palsy |
| 10072042 | Parinaud syndrome |
| 10080905 | Overwear syndrome |
| 10005177 | Blindness cortical |
| 10007748 | Cataract cortical |
| 10070917 | Eccentric fixation |
| 10078228 | Diffuse uveal melanocytic proliferation |
| 10046851 | Uveitis |
| 10072686 | Uveitic glaucoma |
| 10041951 | Staphyloma |
| 10080316 | Tractional retinal detachment |
| 10057884 | Bowman's membrane disorder |
| 10076692 | Anterior segment ischaemia |
| 10086342 | Anterior segment neovascularisation |
| 10002681 | Anterior chamber degeneration |
| 10002683 | Anterior chamber opacity |
| 10061641 | Anterior chamber disorder |
| 10068960 | Narrow anterior chamber angle |
| 10058664 | Anterior chamber crystallisation |
| 10057400 | Anterior chamber pigmentation |
| 10071684 | Anterior chamber collapse |
| 10053781 | Anterior chamber cell |
| 10054774 | Anterior chamber fibrin |
| 10052127 | Anterior chamber flare |
| 10054765 | Anterior chamber inflammation |
| 10079621 | Prostaglandin analogue periorbitopathy |
| 10054045 | Anterior capsule contraction |
| 10088740 | Anterior capsule opacification |
| 10020352 | Holmes-Adie pupil |
| 10018304 | Glaucoma |
| 10018333 | Glaucomatocyclitic crises |
| 10087733 | Glaucomatous optic neuropathy |
| 10088518 | Retrobulbar oedema |
| 10059397 | Antimetropia |
| 10038266 | Refractive amblyopia |
| 10038264 | Refraction disorder |
| 10033683 | Panophthalmitis |
| 10030924 | Optic ischaemic neuropathy |
| 10086436 | Anaemic retinopathy |
| 10038936 | Retinopathy solar |
| 10065373 | Papillophlebitis |
| 10001906 | Amblyopia |
| 10087885 | Triplopia |
| 10003569 | Astigmatism |
| 10013892 | Dyschromatopsia |
| 10008795 | Chromatopsia |
| 10054856 | Pigmentary maculopathy |
| 10052134 | Pigment dispersion syndrome |
| 10035015 | Pigmentary glaucoma |
| 10039677 | Scintillating scotoma |
| 10034962 | Photopsia |
| 10075403 | Superior corneal epithelial arcuate lesion |
| 10078410 | Superior limbic keratoconjunctivitis |
| 10085542 | Superior oblique myokymia |
| 10015995 | Eyelid ptosis |
| 10061312 | Neurological eyelid disorder |
| 10069732 | Neurotrophic keratopathy |
| 10015901 | Exudative retinopathy |
| 10005169 | Blindness |
| 10076381 | Schlemm's canal obstruction |
| 10064133 | Loss of visual contrast sensitivity |
| 10067317 | Oculorespiratory syndrome |
| 10020939 | Hypoaesthesia eye |
| 10049155 | Visual brightness |
| 10047571 | Visual impairment |
| 10047532 | Visual acuity reduced transiently |
| 10047531 | Visual acuity reduced |
| 10003552 | Asthenopia |
| 10030917 | Optic disc drusen |
| 10071196 | Optic disc hyperaemia |
| 10030919 | Optic disc haemorrhage |
| 10057423 | Optic disc telangiectasia |
| 10030923 | Optic discs blurred |
| 10082596 | Optic disc traction syndrome |
| 10057422 | Optic disc vascular disorder |
| 10061321 | Optic disc disorder |
| 10085406 | Peripapillary pigmentation |
| 10053549 | Altered visual depth perception |
| 10030931 | Optic nerve cupping |
| 10061323 | Optic neuropathy |
| 10030936 | Optic nerve infarction |
| 10061322 | Optic nerve disorder |
| 10030941 | Optic nerve sheath haemorrhage |
| 10033712 | Papilloedema |
| 10030910 | Optic atrophy |
| 10076302 | Optic nerve compression |
| 10059081 | Retinal white dots syndrome |
| 10085059 | Retinal occlusive vasculitis |
| 10038845 | Retinal degeneration |
| 10038923 | Retinopathy |
| 10062776 | Retinal drusen |
| 10038891 | Retinal pallor |
| 10077911 | Retinal collateral vessels |
| 10038847 | Retinal deposits |
| 10038867 | Retinal haemorrhage |
| 10038827 | Retinal artery occlusion |
| 10038829 | Retinal artery spasm |
| 10064145 | Retinal aneurysm |
| 10079121 | Retinal aneurysm rupture |
| 10038826 | Retinal artery embolism |
| 10038830 | Retinal artery stenosis |
| 10038831 | Retinal artery thrombosis |
| 10048955 | Retinal toxicity |
| 10061492 | Retinoschisis |
| 10051742 | Retinal infarction |
| 10069652 | Retinal phototoxicity |
| 10071004 | Detachment of macular retinal pigment epithelium |
| 10038853 | Retinal disorder |
| 10064833 | Retinal infiltrates |
| 10038907 | Retinal vein occlusion |
| 10081463 | Retinal vein varices |
| 10038908 | Retinal vein thrombosis |
| 10057429 | Eales' disease |
| 10038899 | Retinal telangiectasia |
| 10038839 | Retinal cyst |
| 10038897 | Retinal tear |
| 10038871 | Retinal ischaemia |
| 10038894 | Retinal pigmentation |
| 10038893 | Retinal pigment epitheliopathy |
| 10052501 | Detachment of retinal pigment epithelium |
| 10090967 | Retinal pigment epithelium change |
| 10062971 | Retinal pigment epithelial tear |
| 10038862 | Retinal exudates |
| 10038886 | Retinal oedema |
| 10038848 | Retinal detachment |
| 10038846 | Retinal depigmentation |
| 10087077 | Retinal microangiopathy |
| 10079569 | Retinal white without pressure |
| 10082240 | Subretinal hyperreflective exudation |
| 10069356 | Subretinal fluid |
| 10062958 | Subretinal fibrosis |
| 10071935 | Subretinal haematoma |
| 10071391 | Retinal fibrosis |
| 10055666 | Retinal neovascularisation |
| 10038903 | Retinal vascular occlusion |
| 10038901 | Retinal vascular disorder |
| 10071246 | Retinal perivascular sheathing |
| 10073562 | Retinal vessel avulsion |
| 10062108 | Retinal vascular thrombosis |
| 10038905 | Retinal vasculitis |
| 10038857 | Retinal dystrophy |
| 10077890 | Retinal thickening |
| 10048896 | Retinal fovea disorder |
| 10038895 | Retinal scar |
| 10063341 | Metamorphopsia |
| 10067557 | Dysmetropsia |
| 10047513 | Vision blurred |
| 10079450 | Visual snow syndrome |
| 10047555 | Visual field defect |
| 10002537 | Anisometropia |
| 10090124 | Binocular visual dysfunction |
| 10002534 | Aniseikonia |
| 10061010 | Binocular eye movement disorder |
| 10034910 | Phlyctenular keratoconjunctivitis |
| 10007749 | Cataract diabetic |
| 10066786 | Diabetic keratopathy |
| 10012692 | Diabetic uveitis |
| 10012667 | Diabetic glaucoma |
| 10012646 | Diabetic blindness |
| 10012689 | Diabetic retinopathy |
| 10012688 | Diabetic retinal oedema |
| 10012661 | Diabetic eye disease |
| 10067567 | Diabetic ophthalmoplegia |
| 10086989 | Idiopathic dilated episcleral vessels |
| 10082001 | Vogt-Koyanagi-Harada disease |
| 10075997 | Idiopathic orbital inflammation |
| 10069664 | Atopic keratoconjunctivitis |
| 10069649 | Atopic cataract |
| 10002535 | Anisocoria |
| 10037532 | Pupillary reflex impaired |
| 10057426 | Pupillary deformity |
| 10037521 | Pupillary disorder |
| 10050003 | Pupillotonia |
| 10028521 | Mydriasis |
| 10027646 | Miosis |
| 10010983 | Corectopia |
| 10037520 | Pupillary block |
| 10078508 | Homonymous diplopia |
| 10051526 | Tolosa-Hunt syndrome |
| 10042441 | Sudden visual loss |
| 10015683 | Exophthalmos |
| 10023683 | Lagophthalmos |
| 10085734 | Oval pupil |
| 10090426 | Outer retinal tubulation |
| 10086195 | Peripheral exudative haemorrhagic chorioretinopathy |
| 10090683 | Kaleidoscope vision |
| 10047589 | Vitamin A deficiency related corneal disorder |
| 10061412 | Vitamin A deficiency eye disorder |
| 10047588 | Vitamin A deficiency related conjunctival disorder |
| 10034960 | Photophobia |
| 10002945 | Aphakia |
| 10063381 | Polypoidal choroidal vasculopathy |
| 10054773 | Aqueous fibrin |
| 10088951 | Trabecular meshwork pigmentation |
| 10042159 | Strabismus |
| 10001912 | Amblyopia strabismic |
| 10047511 | Vision abnormal neonatal |
| 10071129 | Neovascular age-related macular degeneration |
| 10020478 | Hyalosis asteroid |
| 10084478 | Post thrombotic retinopathy |
| 10001914 | Amblyopia tobacco |
| 10063534 | Ocular dysmetria |
| 10067103 | Ocular surface disease |
| 10015916 | Eye disorder |
| 10065700 | Ocular sarcoidosis |
| 10086703 | Ocular cyst |
| 10079891 | Eye haematoma |
| 10012369 | Deposit eye |
| 10030041 | Ocular hyperaemia |
| 10015926 | Eye haemorrhage |
| 10015946 | Eye irritation |
| 10074331 | Cholesterolosis bulbi |
| 10086395 | Ophthalmic artery occlusion |
| 10086406 | Ophthalmic artery aneurysm |
| 10081144 | Ophthalmic artery thrombosis |
| 10061129 | Eye movement disorder |
| 10061137 | Ocular toxicity |
| 10015915 | Eye discharge |
| 10076569 | Eye paraesthesia |
| 10082768 | Hyperaesthesia eye |
| 10082764 | Anaesthesia eye |
| 10000173 | Abnormal sensation in eye |
| 10083006 | Eye infarction |
| 10015907 | Eye allergy |
| 10072139 | Ocular rosacea |
| 10057675 | Posterior segment of eye anomaly |
| 10030875 | Ophthalmoplegia |
| 10065088 | Eye muscle entrapment |
| 10049168 | Ocular myasthenia |
| 10079646 | Eyelash changes |
| 10080075 | Eyelash hypopigmentation |
| 10052143 | Ocular discomfort |
| 10078394 | Eye opacity |
| 10052139 | Eye oedema |
| 10072289 | Eye colour change |
| 10052140 | Eye pruritus |
| 10074349 | Ophthalmic vein thrombosis |
| 10012141 | Deformity of orbit |
| 10031050 | Orbital myositis |
| 10054717 | Disorder of orbit |
| 10031037 | Orbital cyst |
| 10079161 | Orbital compartment syndrome |
| 10031044 | Orbital granuloma |
| 10031051 | Orbital oedema |
| 10031029 | Orbit atrophy |
| 10083565 | Orbital haematoma |
| 10083097 | Orbital space occupying lesion |
| 10085251 | Orbital swelling |
| 10034545 | Periorbital oedema |
| 10073423 | Eye ulcer |
| 10048673 | Tear discolouration |
| 10016760 | Flat anterior chamber of eye |
| 10038011 | Recession of chamber angle of eye |
| 10079171 | Deep anterior chamber of the eye |
| 10057571 | Ocular retrobulbar haemorrhage |
| 10053693 | Excessive ocular convergence |
| 10054716 | Disorder of globe |
| 10014853 | Enophthalmos |
| 10065684 | Atrophy of globe |
| 10089441 | Post-enucleation socket syndrome |
| 10069385 | Ocular ischaemic syndrome |
| 10067776 | Ocular pemphigoid |
| 10015958 | Eye pain |
| 10061127 | Eye degenerative disorder |
| 10053635 | Extraocular muscle disorder |
| 10052896 | Ocular vascular disorder |
| 10087432 | Ophthalmic vascular thrombosis |
| 10066926 | Ocular vasculitis |
| 10071934 | Intraocular haematoma |
| 10015943 | Eye inflammation |
| 10051116 | Foreign body sensation in eyes |
| 10021124 | Hypotony of eye |
| 10075536 | Eye symptom |
| 10015967 | Eye swelling |
| 10059407 | Parophthalmia |
| 10053196 | Eyelid bleeding |
| 10057385 | Eyelid irritation |
| 10083615 | Eyelid calcification |
| 10015999 | Eyelid sensory disorder |
| 10061145 | Eyelid function disorder |
| 10079647 | Eyelid vellus hair changes |
| 10015237 | Erythema of eyelid |
| 10015997 | Eyelid retraction |
| 10078745 | Eyelid myokymia |
| 10076060 | Eyelid myoclonus |
| 10061130 | Eyelid disorder |
| 10060762 | Eyelid erosion |
| 10063692 | Eyelid cyst |
| 10071717 | Eyelid skin dryness |
| 10012493 | Dermatochalasis |
| 10077301 | Cutaneous horn of eyelid |
| 10074620 | Eyelid rash |
| 10005147 | Blepharal pigmentation |
| 10021065 | Hypopigmentation of eyelid |
| 10014194 | Eczema eyelids |
| 10015993 | Eyelid oedema |
| 10069497 | Floppy eyelid syndrome |
| 10005152 | Blepharochalasis |
| 10059208 | Eyelid pain |
| 10015982 | Eyelid degenerative disorder |
| 10064580 | Eyelid exfoliation |
| 10061147 | Eyelid vascular disorder |
| 10064976 | Eyelid haematoma |
| 10052132 | Eyelid margin crusting |
| 10072719 | Eyelid thickening |
| 10042690 | Swelling of eyelid |
| 10051627 | Eyelids pruritus |
| 10030034 | Ocular fistula |
| 10029404 | Night blindness |
| 10001903 | Amaurosis fugax |
| 10005184 | Blindness transient |
| 10049511 | Fuchs' syndrome |
| 10037263 | Pterygium |
| 10020015 | Heterophoria |
| 10010804 | Contact lens intolerance |
| 10075402 | Contact lens acute red eye |
| 10023339 | Keratitis sclerosing |
| 10023353 | Keratoconus |
| 10075189 | Purtscher retinopathy |
| 10020675 | Hypermetropia |
| 10038933 | Retinopathy of prematurity |
| 10057896 | Proliferative vitreoretinopathy |
| 10038934 | Retinopathy proliferative |
| 10065166 | Excessive eye blinking |
| 10052087 | Oscillopsia |
| 10067013 | Normal tension glaucoma |
| 10085732 | Steatoblepharon |
| 10044135 | Toxic cataract |
| 10044245 | Toxic optic neuropathy |
| 10081186 | Central vision loss |
| 10086644 | Central serous chorioretinopathy |
| 10083329 | Foveal degeneration |
| 10080110 | Spontaneous hyphaema |
| 10075690 | Autoimmune uveitis |
| 10071578 | Autoimmune retinopathy |
| 10081652 | Serpiginous choroiditis |
| 10052124 | Meibomian gland discharge |
| 10065062 | Meibomian gland dysfunction |
| 10008388 | Chalazion |
| 10027137 | Meibomianitis |
| 10072716 | Lid sulcus deepened |
| 10024443 | Lid lag |
| 10005159 | Blepharospasm |
| 10005155 | Blepharophimosis |
| 10061842 | Entropion |
| 10042736 | Symblepharon |
| 10014179 | Ectropion |
| 10052123 | Lid margin discharge |
| 10005148 | Blepharitis |
| 10089433 | Blepharokeratoconjunctivitis |
| 10069641 | Blepharosynechia |

**Supplementary Table 2.** Principle of Disproportionality Analysis and Signal Detection Criteria

| **Methods** | **Calculation formula** | **Inclusion standard of positive signal** |
| --- | --- | --- |
| ROR | $ROR=\frac{\left(\frac{a}{c}\right)}{\left(\frac{b}{d}\right)}$ | *a*≥3 and 95%CI>1 |
|  | $SE\left(\ln ROR\right)=\sqrt{\frac{1}{a}+\frac{1}{b}+\frac{1}{c}+\frac{1}{d}}$ |  |
|  | $95\%CI=e^{\ln\left(ROR\right)\pm1.96\sqrt{\frac{1}{a}+\frac{1}{b}+\frac{1}{c}+\frac{1}{d}}}$ |  |
| PRR | $PRR=\frac{\frac{a}{a+b}}{\frac{c}{c+d}}$ | a≥3 and 95%CI>1 |
|  | $SE\left(\ln PRR\right)=\sqrt{\frac{1}{a}-\frac{1}{a+b}+\frac{1}{c}-\frac{1}{c+d}}$ |  |
|  | $95\%CI=e^{\ln\left(PRR\right)\pm1.96\sqrt{\frac{1}{a}-\frac{1}{a+b}+\frac{1}{c}-\frac{1}{c+d}}}$ |  |
| BCPNN |  | 1. No Signal (-): IC_025_  ≤ 0 2. Low Signal (+): 0 < IC_025_ ≤ 1.5 3. Medium Signal (++): 1.5 < IC_025_ ≤ 3 4. High Signal (+++): IC_025_ > 3 |
|  | $E\left(IC\right)=\log_{2}\frac{\left(a+y11\right)\left(a+b+c+d+\alpha\right)\left(a+b+c+d+\beta\right)}{\left(a+b+c+d+\gamma\right)\left(a+b+a1\right)\left(a+c+\beta1\right)}$ |  |
|  | $V(IC)=\frac{1}{(\ln2)^{2}}\left\{\frac{(a+b+c+d)-a+\gamma-\gamma11}{(a+\gamma11)\cdot(1+a+b+c+d+\gamma)}+\frac{(a+b+c+d)-(a+b)+\alpha-\alpha1}{(a+b+\alpha1)\cdot(1+a+b+c+d+\alpha)}+\frac{(a+b+c+d)-(a+c)+\beta-\beta1}{(a+c+\beta1)\cdot(1+a+b+c+d+\beta)}\right\}$ |  |
|  | $\gamma=\gamma11\frac{\left(a+b+c+d+\alpha\right)\left(a+b+c+d+\beta\right)}{\left(a+b+\alpha1\right)\left(a+c+\beta1\right)}$ |  |
|  | $IC-2SD=E\left(IC\right)-2\sqrt{V\left(IC\right)}$ |  |
|  | $Where \alpha1=\beta1=1; \alpha=\beta=2; \gamma11=1$ |  |
| MGPS | $EBGM=\frac{a\left(a+b+c+d\right)}{\left(a+c\right)\left(a+b\right)}$ | EBGM₀₅ > 2 and a>0 |
|  | $EBGM05=e^{\ln(EBGM)-\sqrt[2]{1.64(\frac{1}{a}+\frac{1}{b}+\frac{1}{c}+\frac{1}{d})}}$ |  |

Abbreviation: RR, reporting odds ratio; PRR, proportional reported ratio; BCPNN, bayesian confidence propagation neural network; MGPS, multi - item gamma poisson shrinker; CI, confidence interval; IC, information component.

**Supplementary Table 3.** All positive signal drugs associated with drug-related ocular injuries and their corresponding ATC codes

| **Serial Number** | **Drug Name** | **ATC Code** |
| --- | --- | --- |
| 1 | DUPILUMAB | A16AX;D11AH;R01;R03DX |
| 2 | CICLOSPORIN | L04AD;S01XA |
| 3 | RANIBIZUMAB | S01LA |
| 4 | AFLIBERCEPT | L01XX;S01LA |
| 5 | BIMATOPROST | D11AX;S01EE |
| 6 | FINGOLIMOD | L04AE |
| 7 | INSULIN GLARGINE | A10AE |
| 8 | INSULIN LISPRO | A10A;A10AB;A10AC;A10AD |
| 9 | BOTULINUM TOXIN TYPE A | A01AD;D11AA;D11AX;G04BD;M03AX;N02CX |
| 10 | CENEGERMIN | S01XA |
| 11 | LATANOPROST | S01EE |
| 12 | LIFITEGRAST | S01XA |
| 13 | BEVACIZUMAB | L01FG;S01LA |
| 14 | DOCETAXEL | L01CD |
| 15 | BROLUCIZUMAB | S01LA |
| 16 | BRIMONIDINE | D11AX;S01EA;S01GA |
| 17 | SILDENAFIL | C02KX;G04BE |
| 18 | INSULIN HUMAN | A10A;A10AB;A10AC;A10AD;A10AE;A10AF |
| 19 | TOPIRAMATE | N02CX;N03AX |
| 20 | PENTOSAN POLYSULFATE | B01AB;C05BA;G04BX |
| 21 | PREDNISOLONE | A01AC;A07EA;C05AA;D07AA;H02AB;M02AX;R01AD;S01BA;S02BA;S03BA |
| 22 | TADALAFIL | C02KX;G04BE;G04CX |
| 23 | MACROGOL 400 | A06AD;S01XA |
| 24 | DORZOLAMIDE;TIMOLOL | S01ED |
| 25 | TRIAMCINOLONE | A01AC;C05AA;D07AB;H02AB;R01AD;R03BA;S01BA |
| 26 | MOXIFLOXACIN | D06AX;J01MA;S01AE;S03AA |
| 27 | BELANTAMAB MAFODOTIN | L01FX |
| 28 | AMIODARONE | C01BD |
| 29 | FARICIMAB | S01LA |
| 30 | TRAVOPROST | S01EE |
| 31 | NAPHAZOLINE;PHENIRAMINE | R01AB;S01GA |
| 32 | BRIMONIDINE;TIMOLOL | S01ED |
| 33 | MACROGOL 400;PROPYLENE GLYCOL | S01XA |
| 34 | HYDROXYCHLOROQUINE | L04AX;P01BA |
| 35 | OLOPATADINE | D04A;R01AC;R06AX;S01GX |
| 36 | TIMOLOL | C07AA;N02CX;S01ED |
| 37 | TAMSULOSIN | G04CA |
| 38 | CARMELLOSE | A06AC;D03AX;D09A;S01XA |
| 39 | BRIMONIDINE;BRINZOLAMIDE | S01EC |
| 40 | AVAPRITINIB | L01EX |
| 41 | KETOTIFEN | D04A;D04AA;R01AC;R06AX;S01GX |
| 42 | TAFLUPROST | S01EE |
| 43 | CARMELLOSE;GLYCEROL | R02AX;S01XA |
| 44 | TEPROTUMUMAB | L04AG |
| 45 | VORICONAZOLE | J02AC;S01AX |
| 46 | LOTEPREDNOL | S01BA |
| 47 | OXYMETAZOLINE | D11AX;R01AA;S01GA;S01XA |
| 48 | OCRIPLASMIN | S01XA |
| 49 | CEFUROXIME | J01DC;S01AA |
| 50 | CALCIUM CHLORIDE;MAGNESIUM CHLORIDE;POTASSIUM;SODIUM ACETATE;SODIUM CHLORIDE;SODIUM CITRATE | B05BB;B05CB;B05XA;S01KX;S01XA |
| 51 | BRINZOLAMIDE | S01EC |
| 52 | DEXAMETHASONE;TOBRAMYCIN | S01CA;S02CA;S03CA |
| 53 | NEPAFENAC | S01BC |
| 54 | VERTEPORFIN | S01LA |
| 55 | PROPYLENE GLYCOL | D02AX;S01XA |
| 56 | PILOCARPINE | N07AX;S01EB |
| 57 | SOLIFENACIN | G04BD |
| 58 | NETARSUDIL | S01EX |
| 59 | ALCAFTADINE | S01GX |
| 60 | VIGABATRIN | N03AG |
| 61 | DORZOLAMIDE | S01EC |
| 62 | MELPHALAN | L01AA |
| 63 | SIPONIMOD | L04AE |
| 64 | PEGCETACOPLAN | L04AJ;S01XA |
| 65 | HYPROMELLOSE | R01AX;S01KA;S01XA;V07AY |
| 66 | RAVULIZUMAB | L04AJ |
| 67 | RALOXIFENE | G03XC |
| 68 | INGENOL | D06BX |
| 69 | DIFLUPREDNATE | D07AC;S01BA |
| 70 | GLYCOPYRRONIUM | A01AD;A03AB;D11AA;R03BB;V03AB |
| 71 | HYOSCINE | A03BB;A04AD;N05CM;S01FA |
| 72 | VARDENAFIL | C02KX;G04BE |
| 73 | TELITHROMYCIN | J01FA |
| 74 | TAMOXIFEN | L02BA |
| 75 | ENCORAFENIB | L01EC |
| 76 | KETOROLAC | M01AB;M02AA;S01BC |
| 77 | MINOCYCLINE | A01AB;D06AA;D10AF;J01AA |
| 78 | DEXAMETHASONE;NEOMYCIN;POLYMYXIN B | S01CA;S02CA;S03CA |
| 79 | IVERMECTIN | D11AX;P02CF;P03AX |
| 80 | FLUOCINOLONE ACETONIDE | C05AA;D07AC;S01BA;S02BA |
| 81 | LOTILANER | S01XA |
| 82 | BROMFENAC | M01AB;S01BC |
| 83 | PROXYMETACAINE | S01HA |
| 84 | RIBOFLAVIN | A11HA;S01XA;V91 |
| 85 | ERYTHROMYCIN | D06AX;D10AF;J01FA;S01AA |
| 86 | BESIFLOXACIN | S01AE |
| 87 | LATANOPROST;NETARSUDIL | S01EE |
| 88 | TRALOKINUMAB | D11AH |
| 89 | OFLOXACIN | D06AX;G01AA;J01MA;P01AB;S01AE;S02AA;S03AA |
| 90 | GENTAMICIN | D06AX;J01GB;S01AA;S02AA;S03AA |
| 91 | PHENTERMINE;TOPIRAMATE | A08AA |
| 92 | MERCAPTAMINE | A16AA;D11AX;S01XA;V03AB |
| 93 | MITOMYCIN | L01DC |
| 94 | TETRYZOLINE | R01AA;S01GA |
| 95 | TROPICAMIDE | S01FA |
| 96 | POLYMYXIN B;TRIMETHOPRIM | A07AA;J01EA;S01AA;S03AA |
| 97 | FLUOROMETHOLONE | C05AA;D07AB;S01BA |
| 98 | TIMOLOL;TRAVOPROST | S01ED |
| 99 | AVOBENZONE;HOMOSALATE;OCTISALATE;OCTOCRILENE;OXYBENZONE | D02BA |
| 100 | BINIMETINIB | L01EE |
| 101 | AMLODIPINE;HYDROCHLOROTHIAZIDE;VALSARTAN | C09DX |
| 102 | OXIGLUTATIONE | L03AX;S01KX |
| 103 | HYPROLOSE | S01XA |
| 104 | ETHAMBUTOL | J04AK |
| 105 | BRINZOLAMIDE;TIMOLOL | S01ED |
| 106 | PEGAPTANIB | S01LA |
| 107 | GANCICLOVIR | D06BB;J05AB;S01AD |
| 108 | LATANOPROSTENE BUNOD | S01EE |
| 109 | PARAFFIN, LIQUID;WHITE SOFT PARAFFIN | D02AC;S01XA |
| 110 | POVIDONE-IODINE | A01AB;D08AG;D09AA;D11AC;G01AX;R01AX;R02AA;S01AX |
| 111 | PIMECROLIMUS | D11AH |
| 112 | ACETAZOLAMIDE | C03BA;N03AX;R07A;S01EC |
| 113 | ATROPINE | A03BA;C01CX;S01FA;V03AB |
| 114 | CARMELLOSE;HYPROMELLOSE | S01XA |
| 115 | GATIFLOXACIN | J01MA;S01AE;S03AA |
| 116 | IVABRADINE | C01EB |
| 117 | DEXTRAN;HYPROMELLOSE | S01XA |
| 118 | MIRVETUXIMAB SORAVTANSINE | L01FX |
| 119 | PERFLUOROHEXYLOCTANE | S01KX;S01XA |
| 120 | POLYVINYL ALCOHOL;POVIDONE | S01XA |
| 121 | BACITRACIN | A07AX;D06AX;J01XX;R02AB;S01AA |
| 122 | LOTEPREDNOL;TOBRAMYCIN | S01CA |
| 123 | CYCLOPENTOLATE | S01FA |
| 124 | BENZOYL PEROXIDE | D06BX;D10AE |
| 125 | PHENYLEPHRINE | C01CA;C05AX;R01AA;R01BA;S01FB;S01GA |
| 126 | DEFEROXAMINE | V03AC |
| 127 | PARAFFIN, LIQUID;PETROLATUM | D02AC;S01X |
| 128 | ETHANOL | C01EB;D08AX;R01AX;V03AB;V03AZ;V07AV |
| 129 | COBIMETINIB | L01EE |
| 130 | BIMATOPROST;TIMOLOL | S01ED |
| 131 | HOMOSALATE;OCTINOXATE;OCTISALATE;OXYBENZONE | D02BA |
| 132 | TISOTUMAB VEDOTIN | L01FX |
| 133 | DARIFENACIN | G04BD |
| 134 | ERDAFITINIB | L01EN |
| 135 | AMINOLEVULINIC ACID | L01XD;V04CX |
| 136 | EPINEPHRINE;LIDOCAINE | N01BB |
| 137 | VORETIGENE NEPARVOVEC | S01XA |
| 138 | SATRALIZUMAB | L04AC |
| 139 | NITISINONE | A16AX |
| 140 | CARMELLOSE;GLYCEROL;POLYSORBATE 80 | S01XA |
| 141 | QUININE | M09AA;P01BC;R05X |
| 142 | CLOMIFENE | G03GB |
| 143 | CHLOROQUINE | L04AX;P01BA |
| 144 | HYOSCYAMINE | A03BA |
| 145 | BETAXOLOL | C07AB;S01ED |
| 146 | ADAPALENE;BENZOYL PEROXIDE | D10AD |
| 147 | NATAMYCIN | A01AB;A07AA;D01AA;G01AA;J02AA;S01AA |
| 148 | AZELASTINE;FLUTICASONE | R01AD |
| 149 | CARTEOLOL | C07AA;S01ED |
| 150 | HYDROCORTISONE;NEOMYCIN;POLYMYXIN B | D07CA;S01CA;S02CA;S03CA |
| 151 | TETRACAINE | A01AD;C05AD;D04AB;N01BA;R02AD;S01HA |
| 152 | HYDROCORTISONE;NEOMYCIN | D07CA;D07CB;G01BA;R01AD;S01CA;S02CA;S03CA |
| 153 | RIFABUTIN | J04AB |
| 154 | AVOBENZONE;HOMOSALATE;OCTISALATE;OCTOCRILENE | D02BA |
| 155 | IODINE (131 I) | V09FX;V09XA;V10XA |
| 156 | ETRASIMOD | L04AE |
| 157 | BEPOTASTINE | D04A;R06AX;S01GX |
| 158 | BENZOYL PEROXIDE;CLINDAMYCIN | D10AF |
| 159 | MOXIFLOXACIN;TRIAMCINOLONE | S01CA |
| 160 | ECOTHIOPATE | S01EB |
| 161 | TROSPIUM | A03AB;G04BD |
| 162 | RETIGABINE | N03AX |
| 163 | CHLORPROMAZINE | A04AD;N05AA |
| 164 | CROMOGLICIC ACID | A07EB;D11AH;R01AC;R03BC;S01GX |
| 165 | DINUTUXIMAB | L01FX |
| 166 | PENTOXIFYLLINE | C04AD |
| 167 | PEMIGATINIB | L01EN |
| 168 | AVACINCAPTAD PEGOL | S01XA |
| 169 | CHLORTALIDONE | C03BA |
| 170 | APRACLONIDINE | S01EA |
| 171 | PREDNISOLONE;SULFACETAMIDE | S01CA;S03CA |
| 172 | CHLORAMPHENICOL | A01AB;D06AX;D10AF;G01AA;J01BA;S01AA;S02AA;S03AA |
| 173 | PIPERONYL BUTOXIDE;TANACETUM CINERARIIFOLIUM | P03AC |
| 174 | ASCORBIC ACID;ERGOCALCIFEROL;NICOTINAMIDE;PYRIDOXINE;RETINOL;RIBOFLAVIN;THIAMINE | A11BA |
| 175 | BACITRACIN;NEOMYCIN;POLYMYXIN B | D06AX;S01AA |
| 176 | ACETYLCHOLINE | N07AB;S01EB |
| 177 | PARAFFIN, LIQUID | A06AA;A06AG;D02AC;S01XA;S02DC;V91 |
| 178 | KETOROLAC;PHENYLEPHRINE | S01FB |
| 179 | MACROGOL | A06AD;S01XA |
| 180 | TRIFLURIDINE | L01BC;S01AD |
| 181 | LIGHT LIQUID PARAFFIN;PARAFFIN, LIQUID;POLYSORBATE 80 | S01XA |
| 182 | LEVOBUNOLOL | S01ED |
| 183 | INSULIN BOVINE;INSULIN PORCINE | A10AB;A10AC;A10AD;A10AE |
| 184 | CARBACHOL | N07AB;S01EB |
| 185 | CIDOFOVIR | J05AB |
| 186 | EYE | V91 |
| 187 | INEBILIZUMAB | L04AG |
| 188 | POLYVINYL ALCOHOL | S01XA |
| 189 | SELENIUM SULFIDE | D01AE;D11AC |
| 190 | SULFACETAMIDE | D10AF;D11AC;S01AB |
| 191 | AVOBENZONE;OCTINOXATE;OCTISALATE;OXYBENZONE | D02BA |
| 192 | BOTULINUM TOXIN TYPE B | A01AD;M03AX |
| 193 | CIPROFLOXACIN;DEXAMETHASONE | S01CA;S02CA;S03CA |
| 194 | FLUOCINONIDE | C05AA;D07AC |
| 195 | NAPHAZOLINE | R01AA;S01GA |
| 196 | EPINASTINE | D04A;R06AX;S01GX |
| 197 | HYALURONIDASE | B06AA;S01KX;V03AB;V03AX |
| 198 | HYDROGEN PEROXIDE | A01AB;D08AX;D11AX;N04;S01XA;S02AA;S02D |
| 199 | FLURBIPROFEN | A01AD;M01AE;M02AA;R02AX;S01BC |
| 200 | GLYCEROL;HYPROMELLOSE;MACROGOL | S01XA |
| 201 | PIPERONYL BUTOXIDE;PYRETHRINS | P03AC |
| 202 | PHENTOLAMINE | C04AB;G04BE;V03AB |
| 203 | TITANIUM DIOXIDE;ZINC | D02AB |
| 204 | DEXTRAN;GLYCEROL;HYPROMELLOSE | S01XA |
| 205 | COSMETICS | V07AT |
| 206 | THIORIDAZINE | N05AC |
| 207 | BACITRACIN;POLYMYXIN B | D06AX;S01AA |
| 208 | CAMPHOR | C01EB;D04AX;M02AX;R05CA |
| 209 | FLUORESCEIN;OXYBUPROCAINE | S01JA |
| 210 | VORHYALURONIDASE ALFA | B06AA;V03AX |
| 211 | UNOPROSTONE | S01EE |
| 212 | SAFINAMIDE | N04BD |
| 213 | BENZALKONIUM;LIDOCAINE | A01AB;D04AB;R02AA |
| 214 | LATANOPROST;TIMOLOL | S01ED |
| 215 | GLYCEROL;NAPHAZOLINE | S01GA |
| 216 | DENILEUKIN DIFTITOX | L01XX |
| 217 | RIMEXOLONE | H02AB;S01BA |
| 218 | GLYCEROL;HYPROMELLOSE;MACROGOL 400;TETRYZOLINE;ZINC | S01XA |
| 219 | SODIUM | A12CA |
| 220 | ASCORBIC ACID;BETACAROTENE;BIOTIN;COLECALCIFEROL;NICOTINAMIDE;PANTOTHENIC ACID;PHYTOMENADIONE;PYRIDOXINE;RETINOL;RIBOFLAVIN;SELENIUM;SODIUM;THIAMINE;TOCOPHEROL;UBIDECARENONE;ZINC | A11AA |
| 221 | BORIC ACID;EPHEDRINE;MAFENIDE;TAURINE;ZINC | S01AB |
| 222 | DEXAMETHASONE;MOXIFLOXACIN | S01CA;S03CA |
| 223 | DEXTRAN | B05AA;S01XA |
| 224 | INFIGRATINIB | L01EN |
| 225 | POVIDONE | A01AD;A07BC;B05AA;D08A;S01XA |
| 226 | INDOCYANINE GREEN | V04CX |
| 227 | OMIDENEPAG | S01EX |
| 228 | EFGARTIGIMOD ALFA;VORHYALURONIDASE ALFA | L04AA |
| 229 | AVENA SATIVA | D02WX;N06BX;V90 |
| 230 | NAPHAZOLINE;POLYSORBATE 80 | S01GA |
| 231 | GATIFLOXACIN;PREDNISOLONE | S01CA |
| 232 | PHENYLEPHRINE;TROPICAMIDE | S01FA |
| 233 | SARECYCLINE | J01AA |
| 234 | NEOMYCIN | A01AB;A07AA;B05CA;D06AX;J01GB;R02AB;S01AA;S02AA;S03AA |
| 235 | FLUNISOLIDE | R01AD;R03BA |
| 236 | ASCORBIC ACID;BETACAROTENE;TOCOPHEROL | A11JA |
| 237 | FISH OIL;MINERALS NOS;VITAMINS NOS;XANTOFYL;ZEAXANTHIN | S01XA |
| 238 | BACITRACIN;HYDROCORTISONE;NEOMYCIN;POLYMYXIN B | D07CA;S01CA;S02CA |
| 239 | BENZALKONIUM | A01AB;D08AJ;D09AA;G01AX;G02BB;R01AX;R02AA;S01XA |
| 240 | ASCORBIC ACID;BETACAROTENE;SELENIUM;TOCOPHEROL | A11JB |
| 241 | TRIFLUOPERAZINE | N05AB |
| 242 | METHAZOLAMIDE | S01EC |
| 243 | PRAZIQUANTEL | P02BA |
| 244 | OCTINOXATE;OCTISALATE;ZINC | D02BA |
| 245 | LIDOCAINE;TETRACAINE | N01BB |
| 246 | REVEFENACIN | R03BB |
| 247 | GRAMICIDIN;NEOMYCIN;POLYMYXIN B | D06AX;S01AA;S03AA |
| 248 | GONADORELIN | H01CA;V04CM |
| 249 | MILTEFOSINE | L01XX;P01CX |
| 250 | CAMPHOR;PHENOL | D08AE |
| 251 | CAMPHOR;ETHANOL | M02AX |
| 252 | DIPHENHYDRAMINE;PHENYLEPHRINE | R01BA |
| 253 | LODOXAMIDE | S01GX |
| 254 | GLYCEROL;PROPYLENE GLYCOL | D02AX;S01XA |
| 255 | METHYL AMINOLEVULINATE | D11AX;L01XD |
| 256 | PYRIMETHAMINE;SULFADOXINE | P01BD |
| 257 | HYALURONIDASE, OVINE | B06AA |
| 258 | LIDOCAINE;PHENYLEPHRINE | N01BB;R01AB |
| 259 | CALCIUM CHLORIDE;CARMELLOSE;POTASSIUM;SODIUM CHLORIDE;SODIUM LACTATE | S01XA |
| 260 | EMEDASTINE | D04A;R06AX;S01GX |
| 261 | CHLORMADINONE;ETHINYLESTRADIOL | D10BX;G03AA;G03AB |
| 262 | GLYCEROL;NAPHAZOLINE;ZINC | S01GA |
| 263 | LEBRIKIZUMAB | D11AH |
| 264 | DEXTRAN;MACROGOL 400;POVIDONE;TETRYZOLINE | S01GA;S01XA |
| 265 | XANOMELINE | N07A |
| 266 | CARBOMER | A01AD;C05AX;G02CX;S01XA |
| 267 | PHENDIMETRAZINE | A08AA |
| 268 | SODIUM TETRADECYL SULFATE | C05BB |
| 269 | CERIVASTATIN | C10AA |
| 270 | AVOBENZONE;OCTISALATE;OCTOCRILENE | D02BA |
| 271 | PYRITHIONE | D11AX |
| 272 | CALCIUM CARBONATE;RISEDRONIC ACID | M05BB |
| 273 | ALCLOMETASONE | D07AB;S01BA |
| 274 | NEOMYCIN;POLYMYXIN B | B05CA;D06AX;S01AA;S03AA |
| 275 | STEROIDS | A14A;D07;H02;R03BA;S01BA;S02BA;S03BA;V03A |
| 276 | GLYCINE | A16AA;B05CX;G02CX;N05CM;V06DD |
| 277 | APROTININ;CALCIUM CHLORIDE;FACTOR I (FIBRINOGEN);FACTOR XIII (FIBRIN STABILISING FACTOR);THROMBIN | B02BC |
| 278 | PODOPHYLLOTOXIN | D06BB |
| 279 | TETRYZOLINE;ZINC | S01AX;S01GA |
| 280 | HYDROXYAMFETAMINE;TROPICAMIDE | S01FA |
| 281 | PEGINTERFERON ALFA-2B;RIBAVIRIN | L03AB |
| 282 | CLOCORTOLONE | D07AB |
| 283 | DEXTROMETHORPHAN;DOXYLAMINE | R05DA |
| 284 | BORIC ACID;PILOCARPINE;THIAMINE | S01EB |
| 285 | RETINOL | A11CA;D10AD;R01AX;S01XA |
| 286 | BRILLIANT BLUE G | V04CX |
| 287 | SULFUR | D10AB;D11AC;D11AX;P03AA;V91 |
| 288 | MACITENTAN;TADALAFIL | C02KX |
| 289 | BENZOYL PEROXIDE;ERYTHROMYCIN | D10AF |
| 290 | METHYLSULFONYLMETHANE | M01AX;M02AX |
| 291 | PSEUDOEPHEDRINE;TRIPROLIDINE | R01BA |
| 292 | BROMPHENIRAMINE;DEXTROMETHORPHAN;PSEUDOEPHEDRINE | R05DA |
| 293 | CALCIUM CHLORIDE;POTASSIUM;SODIUM CHLORIDE | B05BB;B05CB;B05XA;D11AX;R01AX |
| 294 | OXYTETRACYCLINE | A01AB;D06AA;G01AA;J01AA;R02AB;S01AA;S02AA |
| 295 | SOMAPACITAN | H01AC |
| 296 | AGALSIDASE ALFA | A16AB |
| 297 | AVOBENZONE;ECAMSULE;OCTOCRILENE;TITANIUM DIOXIDE | D02BA |
| 298 | TRICLOSAN | D08AE;D09AA;D10AX |
| 299 | ALOE VERA;PANTHENOL | D11AX |
| 300 | OXYMETAZOLINE;TETRACAINE | N01BA |
| 301 | CYCLOPENTOLATE;PHENYLEPHRINE | S01FB |
| 302 | ALISKIREN;AMLODIPINE | C09XA |
| 303 | AVOBENZONE;OCTISALATE;OCTOCRILENE;OXYBENZONE | D10AX |
| 304 | DEXTROMETHORPHAN;GUAIFENESIN;PHENYLPROPANOLAMINE | R05FA |
| 305 | CAMPHOR;MENTHOL;SALICYLIC ACID | D02AF;M02AC;R01AX |
| 306 | TRYPAN BLUE | S01KX |
| 307 | AMPRENAVIR | J05AE |
| 308 | MELALEUCA ALTERNIFOLIA | D08AW;V90 |
| 309 | AMBENONIUM | N07AA |
| 310 | DEXAMETHASONE;GENTAMICIN;TETRYZOLINE | S01CA |
| 311 | CYCLOPENTOLATE;PHENYLEPHRINE;TROPICAMIDE | S01F |
| 312 | MINERAL OIL LIGHT;PARAFFIN, LIQUID | S01XA |
| 313 | HYDROCORTISONE;OXYTETRACYCLINE;POLYMYXIN B | D07CA;G01BA;G04BX;S03CA |
| 314 | GUAIFENESIN;PARACETAMOL;PHENYLEPHRINE | N02BE |
| 315 | MECOBALAMIN | B03BA;N07XX |
| 316 | DEXTROMETHORPHAN;PROMETHAZINE | R05DA |
| 317 | MOXIFLOXACIN;NEPAFENAC;PREDNISOLONE | S01CA |
| 318 | METIPRANOLOL | C07AA;S01ED |
| 319 | GALLIUM (67GA) | V09HX |
| 320 | ATROPINE;HYOSCINE;HYOSCYAMINE;PHENOBARBITAL | A03CB |
| 321 | HPV VACCINE | J07BM |
| 322 | EPINEPHRINE;MEPIVACAINE | N01BB |
| 323 | DEXBROMPHENIRAMINE;DEXTROMETHORPHAN;PSEUDOEPHEDRINE | R05DA |
| 324 | BROMPHENIRAMINE | R06AB |
| 325 | BROMFENAC;MOXIFLOXACIN;PREDNISOLONE | S01CA |
| 326 | HOMATROPINE | A03BA;S01FA |
| 327 | METFORMIN;NATEGLINIDE | A10BD |
| 328 | GLIMEPIRIDE;PIOGLITAZONE | A10BD |
| 329 | METHYLSCOPOLAMINE | A03BB;S01FA |
| 330 | TITANIUM | A01 |
| 331 | DICYCLOVERINE;DOXYLAMINE;PYRIDOXINE | A04AD |
| 332 | ASCORBIC ACID;BIOTIN;CHROMIUM;COPPER;FOLIC ACID;MAGNESIUM;MANGANESE;NICOTINAMIDE;PANTOTHENIC ACID;POTASSIUM;PYRIDOXINE;RETINOL;RIBOFLAVIN;SELENIUM;THIAMINE;TOCOPHEROL;VITAMIN B12 NOS;ZINC | A11JB |
| 333 | HYOSCYAMINE;METHENAMINE;METHYLTHIONINIUM;PHENYL SALICYLATE;SODIUM PHOSPHATE | J01XX |
| 334 | ARSENIC | D11AX;L01XX;V03AX;V91 |
| 335 | HYDROCORTISONE;MICONAZOLE | D01AC |
| 336 | DIFLORASONE | D07AC |
| 337 | CETRIMIDE;CHLORHEXIDINE | D08AC |
| 338 | MEDRONIC ACID | M05BA;V04CX;V07AY |
| 339 | BROMPHENIRAMINE;DEXTROMETHORPHAN;GUAIFENESIN;PSEUDOEPHEDRINE | R05FA |
| 340 | BROMFENAC;GATIFLOXACIN;PREDNISOLONE | S01C |
| 341 | RICINUS COMMUNIS | A06AB;D02WB;S01XW;V90 |
| 342 | DEXAMETHASONE;NEOMYCIN | D07CB;R01AD;S01CA;S02CA;S03CA |
| 343 | ALLIUM CEPA;APIS MELLIFICA;ATROPA BELLA-DONNA;AYAPANA TRIPLINERVIS;GELSEMIUM SEMPERVIRENS;PHYTOLACCA SPP.;POTASSIUM DICHROMATE;PULSATILLA VULGARIS;STRYCHNOS NUX-VOMICA | V91 |
| 344 | ETHYLDIHYDROXYPROPYL AMINOBENZOATE;OCTINOXATE;OCTOCRILENE | D02BA |
| 345 | OXYBENZONE | D02BA |
| 346 | SODIUM CHLORIDE;WATER | R01AX;V03AX |
| 347 | DIPIVEFRINE | S01EA |
| 348 | MACROGOL;NAPHAZOLINE | S01GA |
| 349 | POVIDONE;PROPYLENE GLYCOL | S01XA |
| 350 | ACETIC ACID;ALUMINIUM ACETATE | S02DC |
| 351 | MINERAL OIL LIGHT;PETROLATUM;WOOL FAT | D02AX;S01XA |
| 352 | OXYTETRACYCLINE;POLYMYXIN B | D06AA;G01AA;S01AA;S03AA |
| 353 | CENTAUREA CYANUS;EUPHRASIA SPP.;HAMAMELIS VIRGINIANA;MATRICARIA CHAMOMILLA | S01XW;V90 |
| 354 | PROTIRELIN | V04CJ |
| 355 | EDROPHONIUM | V04CX |
| 356 | ADIPIODONE MEGLUMINE;AMIDOTRIZOIC ACID | V08A |
| 357 | ALLIUM CEPA;ASPARAGUS RACEMOSUS;ECLIPTA PROSTRATA;MUCUNA PRURIENS | G04BX;V90 |
| 358 | DEXAMETHASONE;OXYTETRACYCLINE | S01CA |
| 359 | METHYLPREDNISOLONE;NEOMYCIN | D07CA;S03CA |
